# Loss of thymidine phosphorylase activity disrupts adipocyte differentiation and induces insulin-resistant lipoatrophic diabetes

**DOI:** 10.1186/s12916-022-02296-2

**Published:** 2022-03-28

**Authors:** Jérémie Gautheron, Lara Lima, Baris Akinci, Jamila Zammouri, Martine Auclair, Sema Kalkan Ucar, Samim Ozen, Canan Altay, Bridget E. Bax, Ivan Nemazanyy, Véronique Lenoir, Carina Prip-Buus, Cécile Acquaviva-Bourdain, Olivier Lascols, Bruno Fève, Corinne Vigouroux, Esther Noel, Isabelle Jéru

**Affiliations:** 1 Centre de Recherche Saint-Antoine (CRSA), Sorbonne Université-Inserm UMRS_938, 27 rue Chaligny 75571, 12 Paris Cedex, France; 2 Institute of Cardiometabolism and Nutrition (ICAN), CHU Pitié-Salpêtrière - Saint-Antoine, Assistance Publique-Hôpitaux de Paris (AP-HP), 75012 Paris, France; 3 Department of Internal Medicine, Division of Endocrinology and Metabolism, Dokuz Eylul University, 35330 Izmir, Turkey; 4 Department of Pediatrics, Division of Metabolic Diseases, Ege University, 35100 Izmir, Turkey; 5 Department of Pediatrics, Division of Pediatric Endocrinology, Ege University, 35100 Izmir, Turkey; 6 Department of Radiology, Dokuz Eylul University, 35100 Izmir, Turkey; 7 Institute of Molecular and Clinical Sciences, St George’s University of London, London, SW17 0RE UK; 8 Platform for Metabolic Analyses, Structure Fédérative de Recherche Necker, Inserm, US24/CNRS UMS 3633, 75015 Paris, France; 9 Institut Cochin, Université Paris Descartes-CNRS UMR8104, Paris, France; 10 Service de Biochimie et Biologie Moléculaire Grand Est, Hospices Civils, UM Pathologies Héréditaires du Métabolisme et du Globule Rouge, CHU de Lyon, 69500 Bron, France; 11 Laboratoire commun de Biologie et Génétique Moléculaires, Hôpital Saint-Antoine, AP-HP, 75012 Paris, France; 12 Centre National de Référence des Pathologies Rares de l’Insulino-Sécrétion et de l’Insulino-Sensibilité (PRISIS), Service de Diabétologie et Endocrinologie de la Reproduction, Hôpital Saint-Antoine, AP-HP, 75012 Paris, France; 13 Département de Médecine Interne, Centre Hospitalier Universitaire, 67000 Strasbourg, France

**Keywords:** TP, Thymidine phosphorylase, Lipodystrophy, Insulin resistance, Mutation, Adipose stem cell, CRISPR-Cas9, Mitochondria, Oxidative stress, Diabetes, Genetics

## Abstract

**Background:**

Thymidine phosphorylase (TP), encoded by the *TYMP* gene, is a cytosolic enzyme essential for the nucleotide salvage pathway. TP catalyzes the phosphorylation of the deoxyribonucleosides, thymidine and 2′-deoxyuridine, to thymine and uracil. Biallelic *TYMP* variants are responsible for Mitochondrial NeuroGastroIntestinal Encephalomyopathy (MNGIE), an autosomal recessive disorder characterized in most patients by gastrointestinal and neurological symptoms, ultimately leading to death. Studies on the impact of *TYMP* variants in cellular systems with relevance to the organs affected in MNGIE are still scarce and the role of TP in adipose tissue remains unexplored.

**Methods:**

Deep phenotyping was performed in three patients from two families carrying homozygous *TYMP* variants and presenting with lipoatrophic diabetes. The impact of the loss of TP expression was evaluated using a CRISPR-Cas9-mediated TP knockout (KO) strategy in human adipose stem cells (ASC), which can be differentiated into adipocytes in vitro. Protein expression profiles and cellular characteristics were investigated in this KO model.

**Results:**

All patients had *TYMP* loss-of-function variants and first presented with generalized loss of adipose tissue and insulin-resistant diabetes. CRISPR-Cas9-mediated TP KO in ASC abolished adipocyte differentiation and decreased insulin response, consistent with the patients’ phenotype. This KO also induced major oxidative stress, altered mitochondrial functions, and promoted cellular senescence. This translational study identifies a new role of TP by demonstrating its key regulatory functions in adipose tissue.

**Conclusions:**

The implication of TP variants in atypical forms of monogenic diabetes shows that genetic diagnosis of lipodystrophic syndromes should include *TYMP* analysis. The fact that TP is crucial for adipocyte differentiation and function through the control of mitochondrial homeostasis highlights the importance of mitochondria in adipose tissue biology.

**Supplementary Information:**

The online version contains supplementary material available at 10.1186/s12916-022-02296-2.

## Background

Diabetes of Mendelian inheritance constitute a heterogeneous group of conditions that cause hyperglycemia, mainly due to molecular defects in genes that are critical for beta cell or adipocyte development and functions. There is a growing interest in identifying the molecular and cellular mechanisms responsible for these inherited diabetes to improve genetic counseling and personalize treatment [1]. Indeed, knowledge of their genetic cause will assist patient treatment, prognosis, and genetic counseling [2, 3]. Among Mendelian diabetes, the most frequent and well-known subgroup is maturity-onset diabetes of the young (MODY) [4]. Lipoatrophic diabetes (LD) constitute another class of inherited diabetes [5, 6]. These lipodystrophic syndromes are characterized by clinical lipoatrophy due to a defect in adipose tissue storage of triglycerides. This results in ectopic lipid infiltration of non-adipose tissues leading to insulin resistance, increased liver glucose production, hypertriglyceridemia, and liver steatosis [3, 5]. About 30 genes have been implicated in LD, but a majority of cases remains genetically unexplained [5, 6].

In the present study, we identified by whole-exome sequencing (WES) two affected siblings with LD and carrying a homozygous splice-site pathogenic variant in *TYMP*, the gene encoding thymidine phosphorylase (TP). A number of *TYMP* loss-of-function mutations have previously been implicated in Mitochondrial NeuroGastroIntestinal Encephalomyopathy (MNGIE; MIM #603041), a rare metabolic autosomal recessive disease [7–9]. MNGIE is mostly characterized by gastrointestinal and neurological manifestations, including severe cachexia, gastrointestinal dysmotility, peripheral neuropathy, leukoencephalopathy, ophthalmoplegia, and ptosis [10, 11]. The disease is progressively degenerative and usually leads to death in early adulthood [12]. Currently, there are no specific therapies for patients with MNGIE and the disease management aims to treat the symptoms evidenced in each individual [13]. The genetic result obtained in the patients investigated herein was unexpected since classical MNGIE manifestations were absent at referral, though the disease evolved towards a more complex phenotype. A third patient with a similar disease onset and carrying a *TYMP* homozygous missense variant was subsequently investigated.

It is well established that the TP enzyme catalyzes the reversible phosphorylation of the deoxyribonucleosides, thymidine (also known as deoxythymidine) and 2′-deoxyuridine, into thymine and uracil, respectively, accompanied by the release of 2-deoxyribose 1-phosphate [10]. The loss of TP activity due to pathogenic variants results in increased cellular levels of thymidine and 2′-deoxyuridine [14, 15]. Although *TYMP* is a nuclear gene and TP a cytosolic protein, mutations affect mitochondrial DNA and function. High deoxyribonucleoside levels are indeed toxic for mitochondrial DNA (mtDNA) and lead to mitochondrial failure due to a progressive acquisition of secondary mtDNA mutations and mtDNA depletion [16]. This ultimately leads to dysfunction of the respiratory chain, and thus, inadequate cellular energy production. The involvement of TP in LD would imply a key role of this enzyme in adipose tissue physiology. In this regard, the expression of TP in adipocytes remains uncertain. It was reported in one study more than 20 years ago that TP was not expressed in adipose tissue, although the corresponding data obtained by an enzyme immunoassay were not presented [17]. Since then, and despite the presence in expression databases of information arguing for the contrary, the idea of an absence of TP in adipocytes has remained persistent in the literature [7, 10, 18]. This could have led to the disregard of a possible role of TP in adipocytes. Most in vitro models of MNGIE described in the literature have used HEK293 cells, HeLa, or fibroblast cells and have facilitated the understanding of the cellular effect of deoxyribonucleoside pool imbalances [19–21]. However, studies in cellular systems with relevance to the organs affected in MNGIE are still lacking.

The aim of this study was to describe the first reported patients with atypical forms of monogenic LD and carrying homozygous pathogenic variants in *TYMP*. This led us to investigate the role of TP in preadipocytes and adipocytes in vitro. The impact of the loss of TP activity on mitochondrial functions, adipogenesis, and insulin sensitivity, as well as cellular senescence, was evaluated using a CRISPR-Cas9-mediated genome-editing approach.

## Methods

### Genetic analyses

Genomic DNA was extracted from peripheral blood leukocytes using standard procedures.

#### Gene panel

A panel containing the following genes involved in LD was analyzed: *ADRA2A*, *AGPAT2*, *AIRE*, *AKT2*, *BANF1*, *BLM*, *BSCL2*, *CAV1*, *CAVIN1*, *CIDEC*, *DYRK1B*, *EPHX1*, *ERCC3*, *ERCC6*, *ERCC8*, *FBN1*, *INSR*, *LEMD2*, *LIPE*, *LMF1*, *LMNA*, *LMNB2*, *MDM2*, *MFN2*, *MTX2*, *NSMCE2*, *PCNT*, *PCYT1A*, *PIK3R1*, *PLIN1*, *POC1A*, *POLD1*, *POLR3A*, *PPARG*, *PTPN11*, *POMP*, *PSMA3*, *PSMB4*, *PSMB8*, *PSMB9*, *PSMG2*, *OTULIN*, *SLC29A3*, *SPRTN*, *WRN*, and *ZMPSTE24*. Exons and flanking intronic sequences were captured from fragmented DNA with the SeqCapEZ enrichment protocol (Roche NimbleGen, WI, USA) followed by paired-end massively parallel sequencing on a MiSeq platform (Illumina, CA, USA) [22]. Bioinformatic analysis was performed using the Sophia DDM pipeline® (Sophia Genetics, Switzerland).

#### Whole exome sequencing (WES)

Library preparation, exome capture, sequencing, and variant annotation were performed by IntegraGen SA (Evry, France). Genomic DNA was captured using the Twist Human Core Exome Enrichment System (Twist Bioscience, OR, USA) and IntegraGen Custom, followed by paired-end 75 bases massively parallel sequencing on Illumina HiSeq4000. Analysis of exome data was performed using Sirius software (IntegraGen SA). *TYMP* variants were described based on the longest isoform (NM_001953.4) using Alamut 2.11 (Sophia Genetics, Lausanne, Switzerland) and Human Genome Variation Society guidelines.

#### Sanger sequencing

The Big Dye Terminator v3.1 sequencing kit (Thermo Fisher Scientific, MS, USA) was employed after PCR amplification and data were analyzed on a 3500xL Dx device with the SeqScape v2.7 software (Thermo Fisher Scientific).

### Computational analysis of TYMP variants

We evaluated the pathogenicity of *TYMP* variants using bioinformatic tools available online. These bioinformatic tools are not the same for splice site and missense variants, as detailed in supplementary materials for each of the two variants identified (Additional file 1: Table S1).

### Adipose stem cell (ASC) isolation, culture, and adipocyte differentiation

ASC were isolated from surgical samples of subcutaneous abdominal adipose tissue from a 25-year-old healthy woman with a normal body mass index (BMI). Adipose tissue was enzymatically digested with collagenase B (0.2%). After centrifugation, stromal vascular fraction was filtered, rinsed, plated, and cultured in α-MEM with 10% fetal calf serum (FCS), 1% GlutaMAX (#35050061, Thermo Fisher Scientific), 1% Penicillin/streptomycin (PS - 10,000 UI/mL), 1% HEPES, and fibroblast growth factor-2 (FGF-2 -145 nmol/L). After 24 h, only ASC adhered to plastic surfaces, while other cells were removed after culture medium replacement. ASC were maintained in an undifferentiated state in α-MEM supplemented with 10% newborn calf serum (#CA-1151500; Biosera, MI, USA), 1% GlutaMAX, HEPES and P/S, and FGF-2 (145 nmol/L). Adipocyte differentiation was induced by treating 2-day post-confluent cultures with high-glucose (25 mmol/L) DMEM supplemented with 10% FCS, 1% PS, 1 μmol/L dexamethasone (#D4902; Sigma-Aldrich, MI, USA), 1 μM rosiglitazone (#D4902; Sigma-Aldrich), 250 μM 3-isobutyl-1-methyl xanthine (IBMX) (#I7018; Sigma-Aldrich), and 0.17 μmol/L insulin (#I0516; Sigma-Aldrich) for 10 days. The medium was then replaced with high-glucose DMEM supplemented with 10% FCS, 1% PS, 1 μmol/L rosiglitazone, and 0.17 μM insulin and changed to fresh medium every 2 days until the 20th day.

### ASC osteoblastic differentiation

Osteoblast differentiation was induced by treating 2-day post-confluent cultures with α-MEM supplemented with 10% FCS, 1% PS, 1 nmol/L vitamin D3 (#C9756; Sigma-Aldrich), 170 μmol/L ascorbic acid (#A8960; Sigma-Aldrich), and 10 mmol/L β-glycerophosphate (#G9422; Sigma-Aldrich) for 14 days.

### CRISPR/Cas9-mediated deletion of TYMP

The lentiviral plasmid plentiCRISPRv2 was a gift from Zhang lab (Addgene, MA, USA; plasmid #52961) and contains hSpCas9, a guide RNA (gRNA), and a puromycin resistance sequence. The gRNA targeting exon 5 of *TYMP* was designed with a well-recognized tool (http://cistrome.org/SSC) to ensure specificity and high cleavage efficiency. Its sequence was the following: sense 5′ CAGAGATGTGACAGCCACCG 3′; antisense 5′ CGGTGGCTGTCACATCTCTG 3′. The web-based tool, CRISPOR (http://crispor.tefor.net) [23] was used to avoid off-target sequences (Additional file 2: Table S2). Lentiviruses dedicated to TP knockdown were produced by the VVTG platform (Federative Research Institute, Necker, France). ASC were infected with viral particles at a minimal titer of 10^8^ units per mL. 48 h post infection, transduced cells were selected with 0.5 μg/mL puromycin dihydrochloride (#P9620; Sigma-Aldrich). Surviving cells were propagated, and the heterogeneous cell pool was used for experiments. The percentage of on-target recombination including insertions and deletions (indels) in the genomic DNA from this cell population was evaluated by Sanger sequencing of *TYMP* exon 5 followed by analysis using the Synthego web-based tool (https://ice.synthego.com).

### Measurement of TP activity

TP activity was measured in white blood cells using a spectrophotometric method. Whole blood was collected at the time of the medical consultation, not necessary at fasting state, for the patients of family A and their parents. The normal reference range for TP activity was determined in the same laboratory using samples from healthy volunteers. Pellets of white blood cells were first homogenized in lysis buffer (50 mmol/L Tris–HCl, pH 7.2, containing 1% Triton X-100, 2 mmol/L phenylmethylsulfonyl fluoride, and 0.02% mercaptoethanol) and sonicated for 10 s, before centrifugation at 20,000g for 30 min at 4°C. Protein concentration in supernatants was determined according to the bicinchoninic acid method on a multiparametric analyser (Indiko™ Clinical Chemistry Analyze; Thermo Fischer Scientific). The reaction mixture containing 100 μg of protein, 15 mmol/L thymidine in 0.1 mol/L Tris–arsenate, pH 6.5, was incubated at 37°C for 1 h. The reaction was stopped by the addition of 1 mL of 0.3 N NaOH. The amount of thymine formed was measured at 300 nm wavelength and determined based on the 3.4 × 10^3^ L/mol/cm difference in the molar extinction coefficient between thymidine and thymine at alkaline pH. Enzyme activity was expressed as μmol of thymine formed per hour per mg of protein.

### Western blot

Cells were suspended in NP-40 lysis buffer. Thirty micrograms of protein extracts were separated by sodium dodecyl sulfate - polyacrylamide gel electrophoresis (SDS-PAGE), transferred to polyvinylidene difluoride membrane and analyzed by immunoblotting using appropriate antibodies (see below for a detailed list). Western blot quantification was performed in triplicate using Fiji software (Open source), and results were normalized to the tubulin protein levels. Uncropped and unedited Western blots seen in the different figures are available in Additional file 3.

### Oil Red-O staining, image processing, and quantification

Intracellular lipids were stained by Oil Red-O (#O0625; Sigma-Aldrich). Cells were washed with phosphate-buffered saline (PBS) and fixed with 4% paraformaldehyde (PFA) in PBS, for 10 min. Fixed cells were incubated with Oil Red-O solution for 1 h at room temperature and then with DAPI (Thermo Fischer Scientific) for 5 min. Fluorescence images were generated with IX83 Olympus microscope, acquired with Cell-Sens V1.6, and analyzed with FIJI software. Images of 8–10 different areas per condition were visualized by fluorescence microscopy using mCherry filter, followed by computer image analysis using FIJI software. Analysis was performed by threshold converting the 8-bit Red-Green-Blue image into a binary image, which consists only of pixels representing lipid droplets (i.e., red). Importantly, after separation, the binary image was manually compared with the original image for consistency and correct binary conversion. The area occupied by lipid droplets in the image was displayed by FIJI software as surface area in μm^2^ and normalized to cell number by semi-automated counting of DAPI-stained nuclei.

### Seahorse analysis

Measurement of mitochondrial respiration (oxygen consumption rates - OCR) was done using a Seahorse XFe96 BioAnalyser (Agilent Technologies, CA, USA). WT, control, and KO ASC were seeded at an optimized density of 10 000 cells/100 μL/well in a 96-well XFe96 cell culture microplate, incubated 24 h, and equilibrated for 1 h in unbuffered XF assay medium (Agilent Technologies) supplemented with 2 mM glutamine, 10 mM glucose, and 1 mM sodium pyruvate. Successive OCR measurements were performed in each well: 3 basal measurements, 3 measurements following the automated injection of 1 μM oligomycin (ATP synthase inhibitor to measure respiration associated with cellular ATP production), 3 following the injection of 1 μM carbonyl cyanide p-trifluoromethoxyphenyl hydrazone (FCCP) (uncoupling agent to measure the maximal respiration capacity), and 3 following the injection of 1 μM antimycin A (electron transport chain inhibitor to measure non-mitochondrial respiration). The data were normalized to the protein content measured in each well using the bicinchoninic acid assay (BCA; Thermo Fisher Scientific) according to the manufacturer’s instructions.

### Quantification of intracellular triglyceride content

Intracellular lipids were extracted from differentiated ASC using hexane/isopropyl alcohol (3:2). Cells were washed and incubated with hexane/isopropyl alcohol (3:2, vol/vol) using 500 μL per well in 6-well culture plates, in a shaker (80 rpm/minute) at room temperature for 60 min. The content of each well was then transferred into a glass tube for nitrogen evaporation of the organic solvent. After evaporation, lipids were resuspended in isopropyl alcohol and transferred into duplicate 96-well plates for analysis after drying. Triglycerides were measured using the Infinity™ Triglyceride kit (Thermo Fischer Scientific) according to manufacturer’s instructions. The absorbance of each well was measured using a Tecan microplate reader (TECAN, Männedorf, Switzerland) and converted to concentration based on a standard curve. Results were normalized to the cell protein content.

### Oxidative stress and cellular senescence

The oxidation of the fluorogenic probe 2,7-dichlorodihydrofluorescein diacetate (CM-H_2_DCFHDA) (2 μg/mL, #C6827; Sigma-Aldrich) was used to evaluate intracellular levels of reactive oxygen species (ROS) on a 200-plate fluorescence reader (TECAN) at 520–595 nm. The blue staining of β-galactosidase (β-gal) at pH 6 was used as a biomarker of cellular senescence. Cells were fixed with 4% PFA in PBS for 5 min at room temperature. Cells were washed twice with PBS and incubated overnight in fresh SA-β-gal staining solution containing 1 mg/mL of X-gal (5-bromo-4-chloro-3-indolyl-β-Dgalactopyranoside) (#3117073001; Sigma-Aldrich), 5 mmol/L potassium ferrocyanide, 5 mmol/L potassium ferricyanide, 150 mmol/L NaCl, 2 mmol/L MgCl_2_, and 0.4 mmol/L phosphate buffer, at pH 6.0, in darkness at 37°C without CO_2_. For positive staining controls, fixed cells were treated with the same solution, but at pH 4.0. After imaging with an IX83 Olympus microscope, stained cells were resuspended with 2% SDS, scratched, and sonicated. Absorbance (630 nm) was read with a Tecan Infinite 200-plate reader, and the pH 6.0/pH 4.0 staining ratio was calculated.

### Statistics

Data are presented as means ± SD (standard deviation). GraphPad Prism software (CA, USA) was used to evaluate statistical significance. Gaussian distribution was tested with the Kolmogorov–Smirnov test. Multiple comparisons were conducted by one-way analysis of variance (ANOVA) with Bonferroni-test or Kruskal-Wallis test for post hoc analysis. *p* < 0.05 was considered statistically significant.

### Key resources table

**Table:** 

**Reagent type or resource**	**Designation**	**Source and reference**	**Identifiers**	**Additional information**
Adipose stem cells	ASC	Pr. Fève lab at CRSA, Paris	N/A	Female, from subcutaneous abdominal adipose tissue
Antibody	Anti-adiponectin	Thermo Fisher Scientific	Cat# MA1-054	WB (1:1000)
Antibody	Anti-AKT	Cell Signaling Technology	Cat# #9272	WB (1:1000)
Antibody	Anti-C/EPBα	Protein Tech	Cat# 18311-1-1P	WB (1:1000)
Antibody	Anti-ERK	Cell Signaling Technology	Cat# 9102	WB (1:1000)
Antibody	Anti-FAS	Cell Signaling Technology	Cat# 3180	WB (1:1000)
Antibody	Anti-IRΒ	Cell Signaling Technology	Cat# 3025	WB (1:1000)
Antibody	Anti-IRS1	Protein Tech	Cat# 17509-1-AP	WB (1:1000)
Antibody	Anti-leptin	Thermo Fisher Scientific	Cat# PA1-051	WB (1:1000)
Antibody	Anti-osteocalcin	Santa Cruz Biotechnology	Cat# sc-74495	WB (1:1000)
Antibody	Anti-P16	Protein Tech	Cat# 10883-1-AP	WB (1:1000)
Antibody	Anti-P21	Protein Tech	Cat# 10355-1-AP	WB (1:1000)
Antibody	Anti-P53	Abcam	Cat# ab1101	WB (1:1000)
Antibody	Anti-P-AKT	Cell Signaling Technology	Cat# #9271	WB (1:1000)
Antibody	Anti-perilipin	Abcam	Cat# ab3526	WB (1:1000)
Antibody	Anti-P-ERK	Cell Signaling Technology	Cat# 9101	WB (1:1000)
Antibody	Anti-P-P53	Abcam	Cat# ab38497	WB (1:1000)
Antibody	Anti-PPARg	Protein Tech	Cat# 16643-1-AP	WB (1:1000)
Antibody	Anti-Runx2	Protein Tech	Cat# 20700-1-AP	WB (1:1000)
Antibody	Anti-SREBP-1	Santa Cruz Biotechnology	Cat# sc-366	WB (1:1000)
Antibody	Anti-Tubulin	Sigma-Aldrich	Cat# T5168	WB (1:10,000)
Antibody	Anti-TP	GeneTex	Cat# GTX23151	WB (1:1000)
Antibody	Anti-P-Tyr	Santa Cruz Biotechnology	Cat# sc-7020	WB (1:500)
Antibody	Anti-rabbit-HRP	Cell Signaling Technology	Cat# 7074	WB (1:3000)
Antibody	Anti-mouse-HRP	Cell Signaling Technology	Cat# 7076	WB (1:3000)
Recombinant DNA reagent (plasmid)	lentiCRISPR v2	Addgene	Cat# 52961	A gift from Zhang lab
Software algorithm	FIJI software	NIH	N/A	
Software algorithm	Prism	Graphpad Software	N/A	

## Results

### Clinical features of patients

**Fig. 1:**
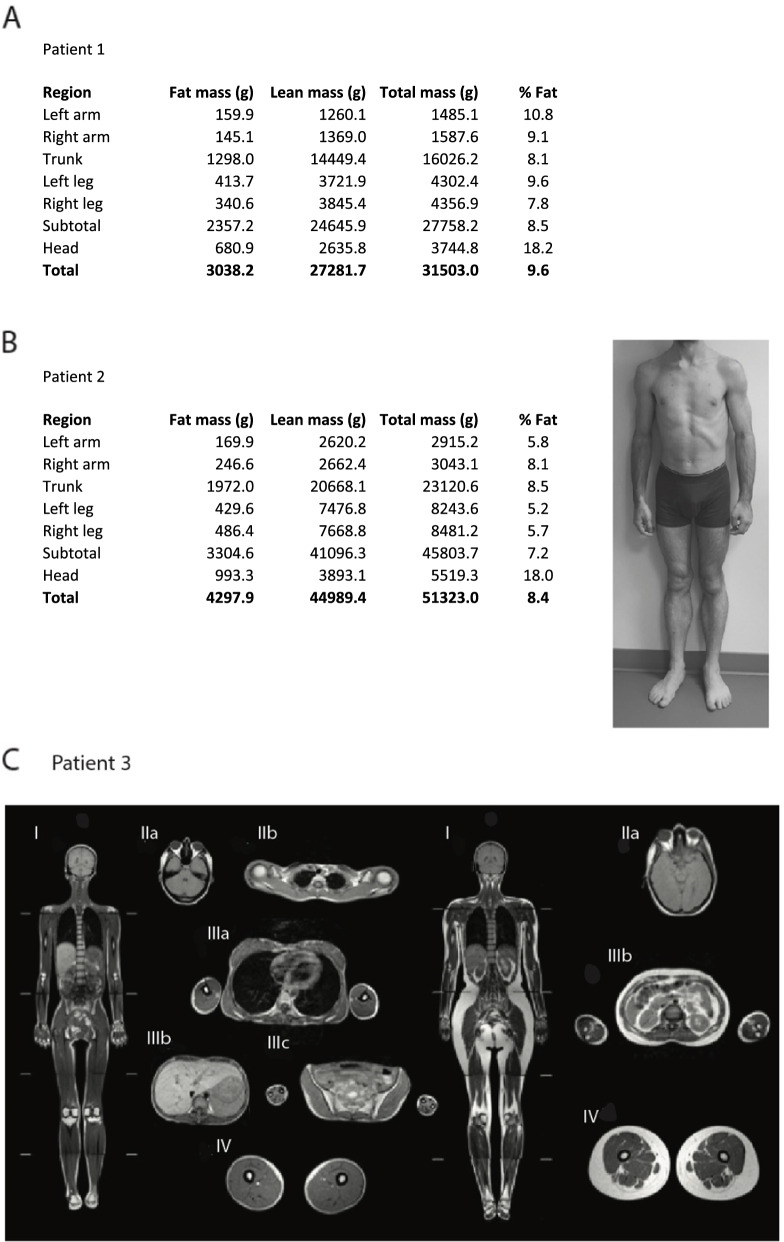
Clinical features and fat distribution in patients. **A** Results of dual-energy X-ray absorptiometry (DXA)-scan showing body composition in patient 1 with total and segmental fat distribution. **B** Left panel: Results of DXA-scan showing body composition in patient 2 with total and segmental fat distribution. Right panel: Front picture of patient 2 (trunk and legs) showing lipoatrophy of the whole body and muscular hypertrophy (upper and lower limbs). **C** Whole body magnetic resonance imaging (MRI) in patient 3 (left panel), as compared to a female control (right panel) showing subcutaneous fat loss in a generalized pattern in patient 3. Whole body pictures (I) correspond to coronal T1-weighted images. Other images are T1-weighted slices. IIa: retroorbital, IIb: supraclavicular, IIIa: trunk, IIIb: upper abdomen showing severe hepatic steatosis in patient 3, IIIc: lower abdomen/pelvic, and IV: proximal lower limbs

The index case from family A, patient 1, was a woman originating from Turkey born from first-cousin healthy parents. She was considered asymptomatic until the age of 6 years, when the parents noted an absence of weight gain, in particular of fat mass. During adolescence, patient 1 and her parents were alerted by the absence of breast or hip enlargement and by the development of hirsutism at the age of 14 years, which led to a first consultation in endocrinology. Generalized lipoatrophy was confirmed by dual X-ray absorptiometry (DXA) with a total fat mass of 9.6%, whereas the mean normal age-matched value is 31.4 ± 8.5% [24] (Fig. 1A and Table 1). Hirsutism was treated by cyproterone acetate and 17-beta estradiol. Patient 1 had a body mass index (BMI) of 12.8 kg/m^2^ at the age of 23 years. Serum leptin levels, which are strongly correlated with total body fat mass, were very low (1.9 ng/mL) and similar to those usually reported in generalized lipodystrophy [25], further confirming the lipoatrophic phenotype. Patient 1 was diagnosed with severe insulin resistant diabetes at the age of 18 years, with high 2-h plasma glucose levels after oral glucose tolerance test (OGTT; 13 mmol/L). Insulin resistance was characterized by *acanthosis nigricans*, as well as by high serum levels of fasting insulin (358.3 pmol/L) and C-peptide (2.2 nmol/L), as well as by the low levels of total serum adiponectin (<0.01 mg/L). The insulinogenic index (IGI), which corresponds to the ratio of insulin concentration at 30 min minus fasting insulin to the difference of glucose at the same time, was calculated after OGTT as a marker of pancreatic beta-cell function. Patient 1 displayed an IGI within the lower normal range (84.3 pmol/mmol; N: 80–180 pmol/mmol) (Table 1). This dynamic test showed a limited insulin secretion capacity in response to glucose challenge, which may reflect either pancreatic exhaustion following insulin resistance or a primitive deficit in insulin secretion. Hypertriglyceridemia was observed (7.6 mmol/L), accompanied by low levels of HDL-cholesterol (0.77 mmol/L). She had hepatomegaly and liver steatosis with elevated levels of aspartate aminotransferase (AST), alanine aminotransferase (ALT), and gamma glutamyl transpeptidase (GGT). Although pubertal development was normal, oligomenorrhoea occurred rapidly after the first menses and progressed to amenorrhea at the age of 16 years. She secondarily developed premature ovarian failure, as revealed by very low levels of oestradiol (<5 ng/L) and elevated levels of gonadotropins (luteinizing hormone (LH): 56 IU/L and follicle-stimulating hormone (FSH): 78.6 IU/L). At the age of 20 years, neurological and gastrointestinal signs evocative of MNGIE syndrome appeared. A demyelinating sensory motor peripheral neuropathy, affecting lower then upper limbs, was confirmed by electromyogram, and led to amyotrophy. Magnetic resonance imaging (MRI) revealed a leukoencephalopathy, associated with unilateral ptosis. Patient 1 also suffered from gastroparesis and abdominal pain due to ileitis. A spontaneous digestive rupture with pneumoperitoneum was followed by a secondary bacterial infection. Patient 1 died at the age of 24 years from the complications of intestinal perforation, in a context of extreme thinness with a BMI of 9 kg/m^2^.

**Table 1:** Clinical and biological features in patients with *TYMP* variants. The age indicated in brackets is the age at diagnosis for the corresponding symptom. Regarding fasting glucose, fasting hyperglycemia is defined by values ranging from 6.1 to 6.9 mmol/L, and diabetes by values ≥ 7 mmol/L. Regarding 2h-OGTT glucose, glucose intolerance is defined by values ranging from 7.8 to 11 mmol/L, and diabetes by values ≥ 11.1 mmol/L. The formula to calculate the insulinogenic index is the following: (insulinemia T30 min – insulinemia T0 min)/(glycemia T30 min – glycemia T0 min) [26]

	Patient 1	Patient 2	Patient 3
**General characteristics**			
Gender	F	M	F
Origin	Turkish	Turkish	Turkish
Age (years)	deceased at the age of 24	27	20
Height (m)	1.63	1.70	1.60
Weight (kg)	34	53	37.5
Body mass index (kg/m^2^)	12.8	18.3	14.6
Age at first symptoms (years)	14	18	15
**Lipodystrophic signs**			
Generalized lipoatrophy	yes (14 years)	yes (18 years)	yes (13 years)
Muscular hypertrophy	no	yes	yes
% of total body fat mass - DXA	9.6	8.4	nd
Leptin levels (ng/mL)	1.9	0.5	0.53
**Gynecological features**			
Hirsutism	yes	na	yes
Amenorrhea	yes	na	yes
**Glucose homeostasis**			
Fasting Insulin (pmol/L) (N < 70 pmol/L)	358.3	519.4	530.6
*Acanthosis nigricans*	yes	yes	yes
Diabetes	yes (18 years)	no	yes (16 years)
Fasting glucose (N: 4.1-6.1 mmol/L)	5.2	4.2	14.2
2h-OGTT glucose (N: ≤ 7.8 mmol/L)	13	10.9	nd
Insulinogenic index (N: 80-180 pmol/mmol)	84.3	110.1	nd
Fasting C-peptide (N: 0.26-0.99 nmol/L)	2.2	3.3	nd
HbA1c (N <6%)	5	4.9	8.4
Adiponectin levels (normal values: 2-14 mg/L)	< 0.01	0.37	nd
**Liver manifestations**			
Hepatomegaly	yes	yes	yes
Liver steatosis	yes	yes	yes
AST / ALT levels (IU/L) (N: <40 IU/L)	142 / 52	100 / 134	50/40
GGT (IU/L) (N: 8-44 IU/L)	77	65	142
**Dyslipidemia**			
Triglycerides levels (N: <1.7 mmol/L)	7.6	2.2	28.1
HDL-cholesterol (N: >1 mmol/L)	0.77	0.65	0.33
**Neurological signs**			
Clinical demyelinating sensory motor peripheral neuropathy	lower and upper limbs (20 years)	no	lower and upper limbs (16 years)
Electromyogram abnormalities	yes	yes	yes
Leukoencephalopathy	yes	no	yes
Ptosis	yes, unilateral	no	
Muscular atrophy	yes		
**Gastrointestinal signs**			
Gastroparesis	yes (20 years)	no	yes (16 years)
Abdominal pain	yes	no	yes
Diarrhea	no	no	yes
**Other clinical signs**			
Hypogammaglobulinemia	yes	no	yes

*ALP* Alkaline phosphatase, *ALT* Alanine aminotransferase, *AST* Aspartate aminotransferase, *DXA* Dual-energy X-ray absorptiometry, *GGT* Gamma glutamyl transpeptidase, *na* Not applicable, *nd* Not determined, *N* Normal value, *OGTT* 75g oral glucose tolerance test

Her brother aged 27 years, patient 2, also had an insulin-resistant lipoatrophic phenotype diagnosed during familial investigations at the age of 18 years. Generalized lipoatrophy was confirmed by DXA with a total fat mass of 8.4%, accompanied by very low leptin levels (0.5 ng/mL) (Fig. 1B). This morphological trait had not attracted attention until that time, unlike the muscular hypertrophy, which was clearly apparent (Fig. 1B). He had insulin resistance, as assessed by high fasting insulin (519.4 pmol/L) and C-peptide levels (3.3 nmol/L) (Table 1). His fasting glucose values and glycated hemoglobin (HbA1c) level remained in the normal range, but OGTT revealed impaired glucose tolerance (Table 1). The IGI value was within the lower normal range (110.1 pmol/mmol). He had mild hypertriglyceridemia (2.2 mmol/L) associated with low HDL-cholesterol levels (0.65 mmol/L). Hepatomegaly was associated with an elevation of liver enzymes (AST, ALT, and GGT) (Table 1) and liver ultrasound revealed liver steatosis. Besides electromyogram abnormalities, this patient did not present any neuro-gastrointestinal manifestations.

In the framework of the European Consortium of Lipodystrophies (ECLip), we identified another Turkish patient with a complex form of LD and carrying a biallelic *TYMP* variant. Patient 3 from family B, born from consanguineous healthy parents, is a 20-year-old woman. She was born with normal adiposity but started to lose subcutaneous fat from her limbs during late childhood. This lipoatrophy became generalized in a few years, and the diagnosis of generalized lipoatrophy was made at the age of 13 years (Table 1). Lipoatrophy was confirmed by whole-body MRI showing a lack of sub-cutaneous and visceral adipose tissue (Fig. 1C). Her BMI was 14.6 kg/m^2^ at the age of 18 years and her serum leptin levels very low (0.5 ng/mL). She was diagnosed at the age of 16 years with severe insulin-resistant diabetes with elevated fasting insulinemia (530.6 pmol/L), *acanthosis nigricans*, fasting hyperglycemia (14.2 mmol/L), and elevated levels of HbA1c (8.4%). She had severe hypertriglyceridemia (28.1 mmol/L) and low HDL-cholesterol (0.33 mmol/L) diagnosed at the age of 17 years. As shown by whole-body MRI (Fig. 1C), she had hepatomegaly and liver steatosis with elevated levels of AST, ALT, and GGT (Table 1). Gastroparesis, demyelinating sensory motor peripheral neuropathy, and leukoencephalopathy occurred at the age of 16 years, 3 years after the onset of the lipoatrophic phenotype.

### Characterization of TYMP variants

**Fig. 2:**
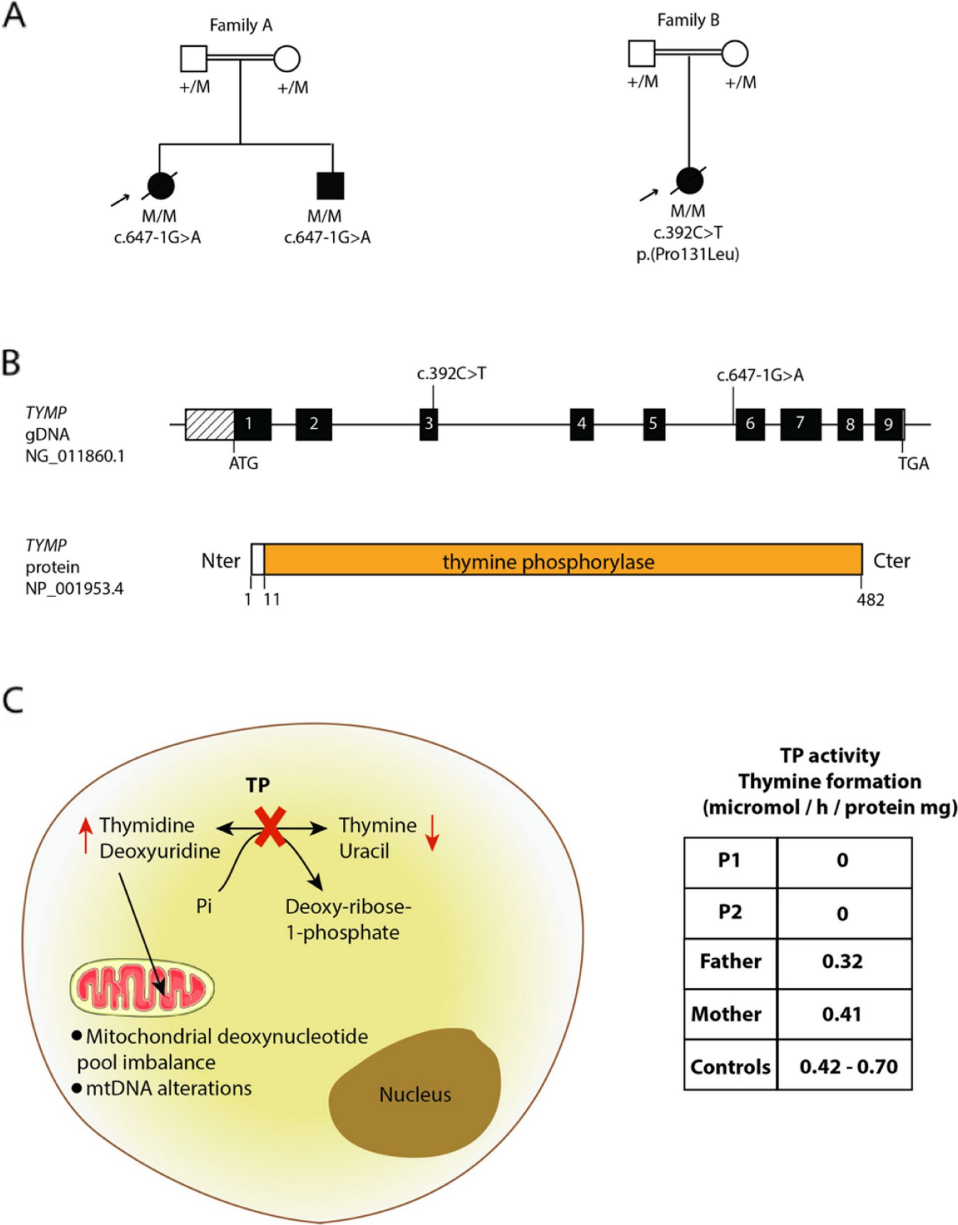
*TYMP* homozygous pathogenic variant in a newly-characterized lipoatrophic diabetes. **A** Genealogical trees and segregation analysis for the *TYMP* variants in the two families investigated herein. Arrows indicate probands. +, normal allele; M, mutant allele. **B** Top panel: schematic representation of *TYMP* genomic sequence displaying the location of the variants identified. Bottom panel: schematic representation of thymidine phosphorylase (TP) protein sequence comprising 482 amino acids. The prediction of protein domain organization was based on UniProt database (protein reference: P19971). **C** Left panel: Schematic drawing showing the functional impact of *TYMP* pathogenic variants. Right panel: Biochemical measurement of TP activity in family A (patients 1 and 2, and their two parents). The enzyme activity was evaluated by an endpoint determination of the thymine formed after 1 h incubation of leukocytes in the presence of an excess of its thymidine substrate

At the time of genetic diagnosis, the two patients from family A displayed insulin resistance and lipoatrophy. The disease of the index case was not explained by variants in genes known to be involved in LD, as assessed by the analysis of a panel of genes used in routine genetic diagnosis. Whole exome sequencing (WES) was carried out in this consanguineous family and led to the identification of a homozygous variant affecting a consensus splice site in the intron 5 of *TYMP*: c.647-1G>A (NM_001953.4). This variant was present in the homozygous state in the two affected children and in the heterozygous state in both parents, who were asymptomatic (Fig. 2A, B). Genotypes were confirmed by Sanger sequencing. To the best of our knowledge, this variant had never been reported before. According to the American College of Medical Genetics and Genomics (ACMG) criteria [27], this variant can be classified as “pathogenic.” It is a variant affecting a canonical splice site, in a gene for which loss-of-function is a known mechanism of disease. Several additional lines of evidence supported the causal role of the variant in the disease phenotype. This variant was absent from databases reporting variants from the general population (gnomAD, ExAC). We evaluated its pathogenicity using several bioinformatic tools available online (spliceAI, SPiP, CADD), which were all in favor of a deleterious effect (Additional file 1: Table S1). No alternative molecular etiology was retained to explain the disease phenotype, when considering the different inheritance modes compatible with the familial history (de novo, compound heterozygous, and homozygous variants). The impact of the c.647-1G>A variant on TP enzyme activity was further confirmed by a biochemical assay evaluating the level of thymine formation in leukocytes, in the presence of an excess of thymidine. TP activity was completely abrogated in leukocytes of patients 1 and 2 as compared to controls, and reduced in their parents who are heterozygous carriers (Fig. 2C). Plasma thymidine and deoxyuridine levels measured in patient 2 were also increased to 17.9 μmol/L and 9.6 μmol/L, respectively, as compared to healthy unaffected controls (*N* < 0.05 μmol/L). Consistent with the diagnosis, elevated levels of serum lactate were observed in patient 1 and patient 2 (2.87 mmol/L and 4.18 mmol/L, respectively; *N* 0.5–2.2 mmol/L). Levels of serum lactate dehydrogenase were also increased in these two patients (325 IU/L and 256 IU/L; *N* 120–246 IU/L).

A missense variant located in *TYMP* exon 3, c.392C>T p.(Pro131Leu), was identified in the homozygous state in patient 3 from family B and in the heterozygous state in her two parents, who were asymptomatic (Fig. 2A, B). This variant could be classified as “likely pathogenic” according to the ACMG classification. This variant is absent from databases reporting variants from the general population. Multiple lines of computational evidence (SIFT, Polyphen-2, REVEL, CADD) support a pathogenic effect for this variant (Additional file 1: Table S1). Another missense pathogenic variant, p.(Pro131Ser), was previously reported at the same position in MNGIE [9], further arguing for a deleterious effect. Consistently, plasma thymidine and deoxyuridine levels were elevated in patient 3: 0.17 μmol/L, 0.44 μmol/L, respectively. This patient also displayed elevated levels of serum lactate (2.5 mmol/L; *N* 0.5–1.6 mmol/L) and serum lactate dehydrogenase (212 IU/L; *N* 98–192 IU/L).

### Disruption of adipocyte differentiation by TP knockout in adipose stem cells (ASC)

**Fig. 3:**
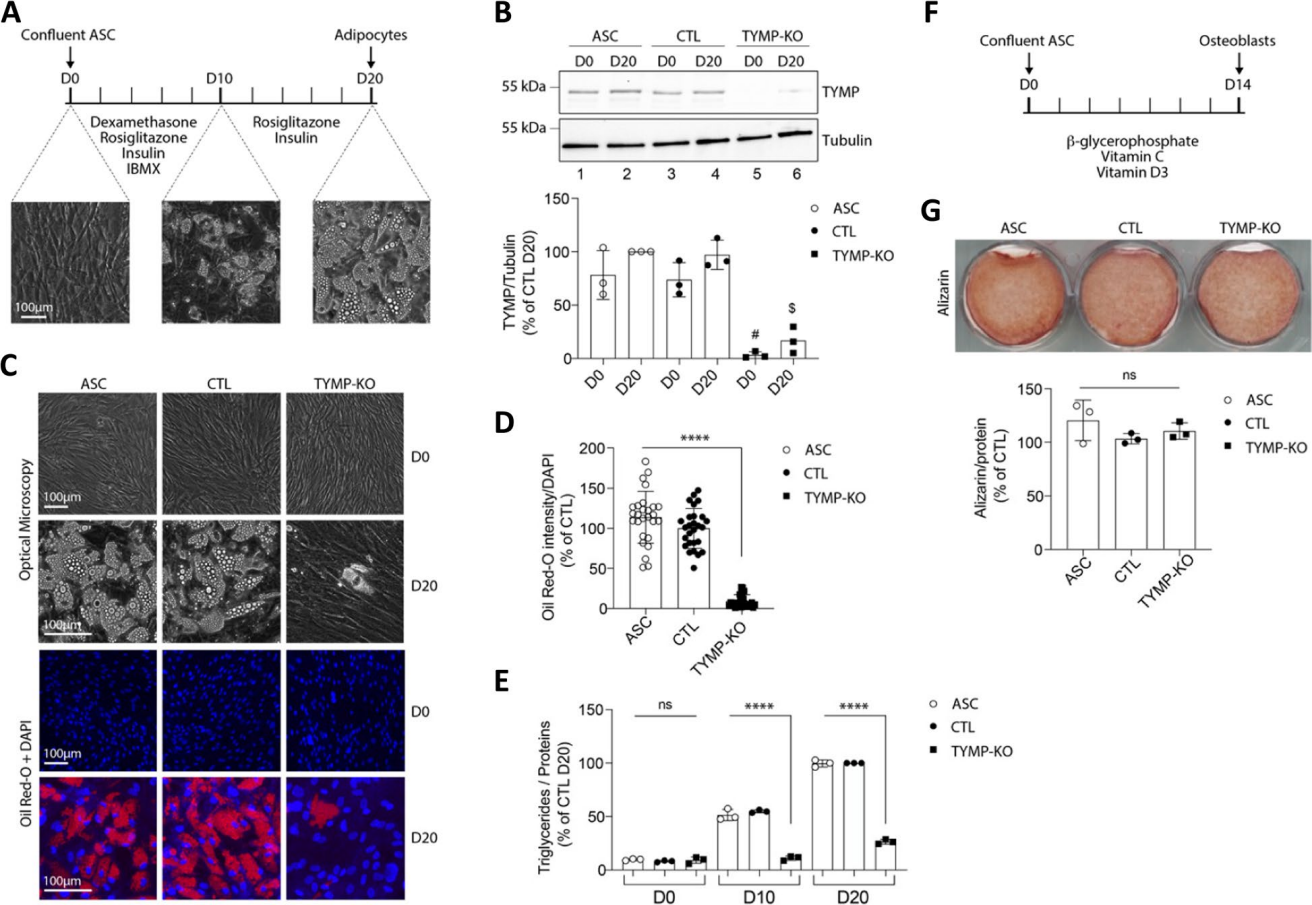
TP deficiency suppresses adipocyte but not osteoblast differentiation of ASC. Data were obtained in ASC, ASC with a CRISPR-Cas9-mediated TP-knockout (KO), and ASC transduced with a Cas9/scramble gRNA plasmid corresponding to control (CTL) cells. **A** Timeline representation of the ASC differentiation process using a hormonal cocktail. IBMX: 3-isobutyl-1-methylxanthine; D0: day 0; D10: day 10; D20: day 20. **B** Thymidine phosphorylase (TP) expression in ASC during adipocyte differentiation and validation of TP KO in ASC at D0 and at D20. Numbers on the left correspond to molecular weight markers (kDa). Western blot images are representative of three independent experiments. **C** Adipocyte differentiation assessed by Oil Red-O lipid staining. ASC pre-adipocytes were studied during adipocyte differentiation for 20 days. First and second lines: representative pictures of cell dishes by optical microscopy. Images are representative of five independent experiments. Third and fourth lines: representative images of fluorescence microscopy after staining of intracellular lipids (Oil Red-O, red) and nuclei (DAPI, blue). Images are representative of five independent experiments. **D** Quantification of Oil Red-O fluorescence normalized to DNA content (DAPI). Results are expressed as means ± SEM of five independent experiments. **E** Evolution of intracellular triglyceride content during in vitro adipocyte differentiation. Triglycerides were measured at D0, D10, and D20 in ASC, CTL, and TP KO cells. Measurements are representative of three independent experiments. **F** Timeline representation of the ASC differentiation process in osteoblasts using a hormonal cocktail. **G** Pictures of dishes stained by Alizarin-red S. Images are representative of three independent experiments. The ratio of Alizarin/protein was calculated after cell lysis and protein extraction. ****p < 0.0001. *p*-values were determined by analysis of variance (ANOVA) with Kruskal-Wallis post hoc multiple comparison test; n.s., non-significant

Although TP was said to be absent in adipocytes based on a study published 25 years ago [17], current expression databases report that adipose tissue is one of the tissues with the highest TP expression level (Additional file 4: Fig. S1). To better address this issue, human ASC were chosen as a cellular model due to their ability to differentiate into mature adipocytes after stimulation in vitro (Fig. 3A). First, we confirmed TP expression in wild-type undifferentiated ASC (day 0–D0) and we observed a slight increase in TP expression during adipocyte differentiation (Fig. 3B; compare lines 1 and 2, around 45% increase). Since the homozygous splice-site variant present in patients results in a complete loss of enzymatic activity, we can mimic this effect by inhibiting the gene expression with a CRISPR-Cas9-mediated knockout (KO) approach. A custom-designed guide RNA (gRNA)/Cas9 expression vector targeting the fifth exon of *TYMP* was used (Additional file 2: Table S2). The efficiency of TP KO was confirmed by Western blot analysis, which led to a complete loss of TP expression at D0 (Fig. 3B; compare lines 1 and 5). In addition, Sanger sequencing of *TYMP* exon 5 in genomic DNA from KO ASC cells revealed a high level of on-target indels with 40% of insertions and 51% of deletions (Additional file 4: Fig. S2). ASC infected with a Cas9/scramble gRNA plasmid were used as a control (CTL). The efficiency of adipocyte differentiation in control ASC was confirmed by progressive lipid accumulation, as revealed both by the appearance of refractive droplets in optical microscopy, and by an increase in Oil Red O staining, which is a marker of intracellular lipids. Wild type (WT) and control ASC differentiated into adipocytes within 20 days (D20) and displayed strong accumulation of lipid droplets in the cytoplasm (Fig. 3C and D). This was confirmed by measurement of the intracellular triglyceride content (Fig. 3E). In contrast, TP KO led to strong and significant decrease in lipid droplet formation (*p* < 0.0001) (Fig. 3C, D) and triglyceride content (*p* < 0.0001) (Fig. 3E).

Notably, ASC correspond to a multipotent stem cell population displaying the ability to be chemically orientated towards adipogenic, as well as osteogenic and chondrogenic cellular phenotypes [28]. Considering that TP is also expressed in bone [29, 30], we took advantage of this ASC property to test the specificity of the loss of adipocyte differentiation. TP KO cells were directed towards osteoblastic lineage using an appropriate hormonal cocktail (Fig. 3F). The capacity for osteoblastic differentiation was assessed by alizarin red S staining, which is a marker of calcium deposits. Staining intensity was similar in WT, CTL, and TP KO cells (Fig. 3F and G). Moreover, two markers of osteoblastic differentiation, namely runx2 and osteocalcin, were investigated on Western blots, and no significant difference was found between WT, CTL, and TP KO cells (Additional file 4: Fig. S3). These results argue against a global defect in the differentiation capacity of ASC and suggest that adipocytes are more prone to dysfunction than osteocytes upon TP deficiency.

#### Alteration of adipocyte function by TP knockout in ASC

**Fig. 4:**
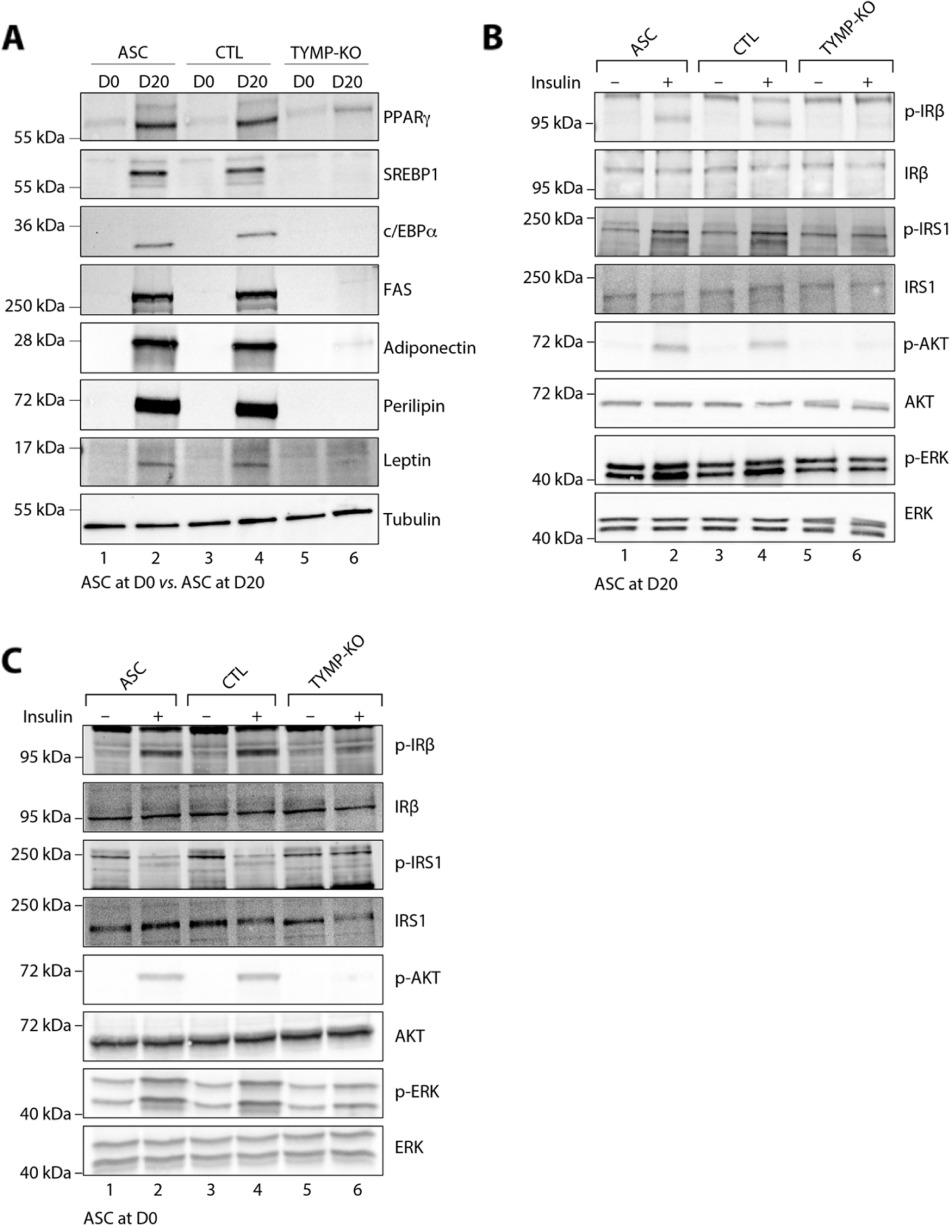
TP deficiency alters adipocyte function of ASC. Data were obtained in ASC, ASC with a CRISPR-Cas9-mediated TP-knockout (KO), and ASC transduced with a Cas9/scramble gRNA plasmid corresponding to control (CTL) cells. **A** Protein expression of adipocyte markers obtained by Western blotting during in vitro adipocyte differentiation of ASC cells at D0 (undifferentiated state) and D20 (day 20 after differentiation onset). Numbers on the left correspond to molecular weight markers (kDa). Western blot images are representative of three independent experiments. PPARγ: peroxisome proliferator-activated receptor-gamma; C/EBPα: CCAAT/enhancer-binding protein-alpha; SREBP-1c: sterol regulatory element-binding protein-1c; FAS: fatty acid synthase. **B** Activation of insulin signaling in adipocytes after 20 days of adipocyte differentiation. ASC, CTL, and TP KO cells were deprived of serum for 6 h, stimulated with 20 nM insulin for 5 min or left untreated, and subjected to immunoblotting with antibodies against total and phospho-insulin receptor β-subunit (IRβ), insulin receptor substrate-1 (IRS1), AKT, and extracellular-regulated kinase (ERK)1/2. Numbers on the left correspond to molecular weight markers (kDa). Western blot images are representative of three independent experiments. **C** Activation of insulin signaling in ASC before differentiation at D0. Same as in **B** in undifferentiated ASC. Western blot images are representative of three independent experiments

The effect of TP KO on adipocyte was first evaluated by an expression study of adipogenic and mature adipocyte markers during the differentiation process. As compared to WT and control cells submitted to in vitro adipocyte differentiation, TP KO cells displayed a sharp decrease in expression of adipogenic markers, including the transcription factors peroxisome proliferator-activated receptor gamma (PPARγ), CCAAT/enhancer-binding protein-alpha (C/EBPα), and SREBP1c at D20 (Fig. 4A; compare line 6 with lines 2 and 4). The expression of mature adipocyte markers, such as fatty acid synthase (FAS), perilipin, adiponectin, and leptin was also sharply decreased in TP KO cells (Fig. 4A and Additional file 4: Fig. S4).

We next investigated the effect of TP loss on insulin sensitivity. In WT and control adipocytes (D20) stimulated with insulin, Western blot analysis revealed a strong increase in the phosphorylation of several signaling intermediates from the mitotic and metabolic pathways including insulin receptor β subunit (IRβ), insulin receptor substrate-1 (IRS1), AKT, and extracellular-regulated kinase (ERK) (Fig. 4B and Additional file 4: Fig. S5A). In contrast, the TP KO cells at D20 were resistant to insulin, as shown by the lack or strong decrease in the phosphorylation of these intermediates upon insulin stimulation (Fig. 4B and Additional file 4: Fig. S5A). Of note, the defect in insulin sensitivity seen in the TP KO cells at D20 was already present at the stage of pre-adipocytes at D0 (Fig. 4C and Additional file 4: Fig. S5B), showing that insulin resistance is not only due to the defect in adipocyte differentiation.

### Alteration of mitochondrial functions and promotion of oxidative stress by TP KO

**Fig. 5:**
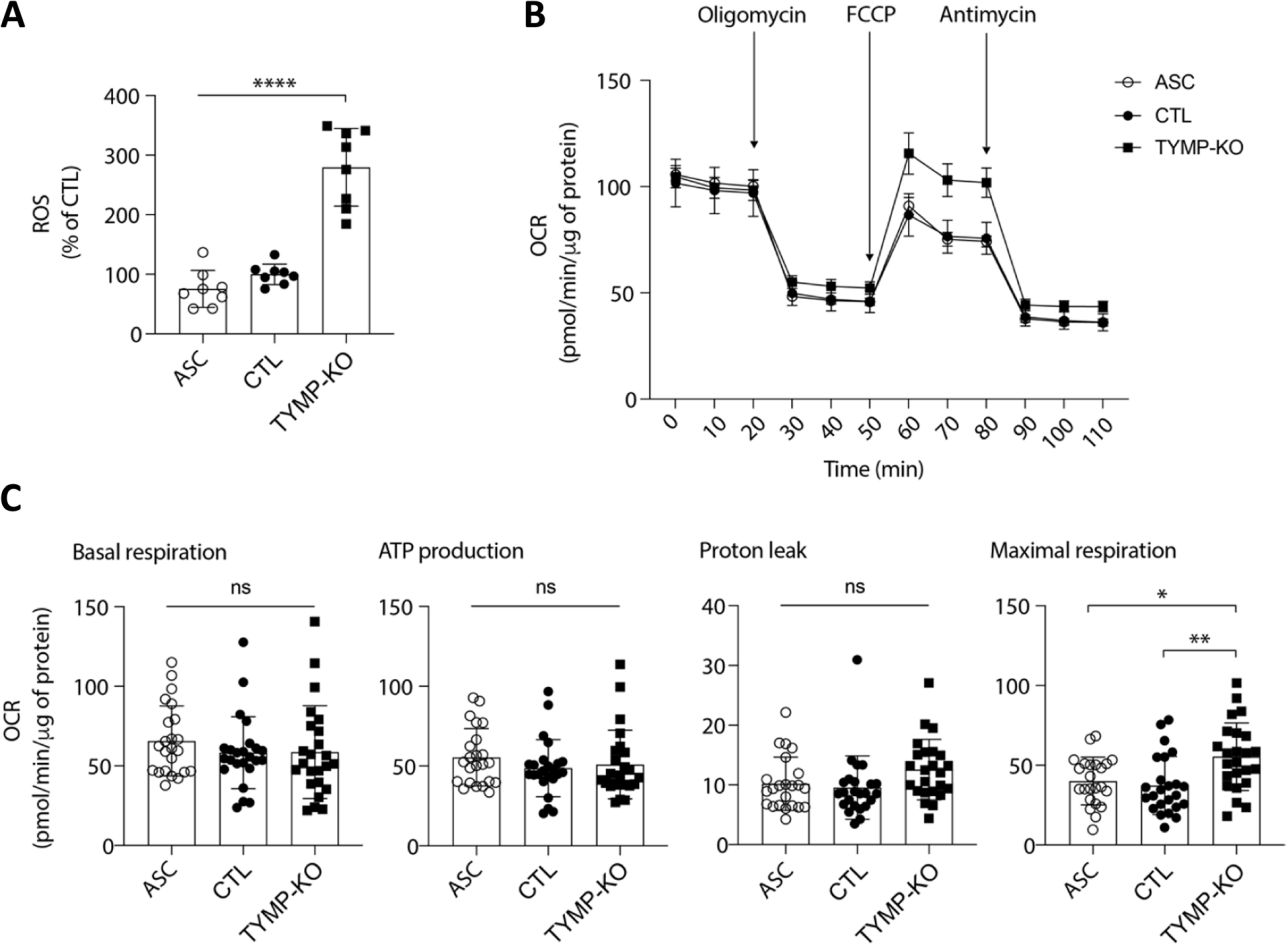
TP deficiency increases ROS levels and mitochondrial respiration in ASC cells. Data were obtained at D0 in undifferentiated ASC, ASC with a CRISPR-Cas9-mediated TP-knockout (KO), and ASC with a Cas9/scramble gRNA KO corresponding to control (CTL) cells. Differences between the three cell lines were determined by analysis of variance (ANOVA) with Bonferroni’s post hoc multiple comparison test. All results are expressed as means ± SEM of five independent experiments. **A** Reactive oxygen species (ROS) production assessed by oxidation of 5-6-chloromethyl-2,7-dichlorodihydro-fluorescein diacetate (CM-H_2_DCFDA) in ASC. Results were normalized to DNA content measured by DAPI. ****p < 0.0001. **B** The respiratory flux profiles of cells were determined using a Seahorse Bioanalyzer as described in the “Methods” section. Data are from six replicates and are a compilation of three independent experiments. **C** Quantification of bio-energetic parameters including oxygen consumption rate (OCR) associated to basal respiration, ATP-linked respiration, proton leak, and maximal respiration capacity. *p < 0. 05, **p < 0. 01, n.s., non-significant

To gain further insight into the mechanisms whereby TP controls adipogenesis, we examined mitochondrial activity and ROS production in TP KO cells at D0. Indeed, as a consequence of playing a central role in cellular energy metabolism, mitochondria produce ROS as a by-product of respiration. Several reports indicated that mutations in almost all mtDNA-encoded genes enhance ROS generation [31]. In addition, it was previously shown that stress-induced mitochondrial ROS (mROS) are anti-adipogenic signaling molecules [32]. We therefore wondered whether TP deficiency, which induces mtDNA alterations [10], promotes ROS production. Lysates of TP KO cells indeed displayed higher levels of ROS, compared to either WT or control ASC (*p* < 0.0001) (Fig. 5A). Since mROS production is linked to the functioning of the respiratory chain, mitochondrial respiration tests were performed. TP KO cells had a basal level of mitochondrial respiration similar to that of WT ASC and control cells (Fig. 5B, C). To distinguish oxygen consumption devoted to ATP synthesis from that due to the natural proton leak across the inner mitochondrial membrane, we added the ATP synthase inhibitor oligomycin, which did not induce any change in oxygen consumption in TP KO cells (Fig. 5B, C). However, the addition of the carbonylcyanide-p-trifluoromethoxy-phenylhydrazone (FCCP) protonophore, which leads to a rapid consumption of oxygen without the generation of ATP, showed that TP KO cells had a markedly higher maximum respiratory rate than ASC and control cells (*p* < 0.001). This increase in mitochondrial respiration capacity was consistent with the high levels of ROS production. Altogether, these data underline the functional link between TP dysfunction and mitochondrial-induced oxidative stress, which may partly contribute to the defect in adipogenesis.

### Promotion of cellular senescence by TP KO in ASC

**Fig. 6:**
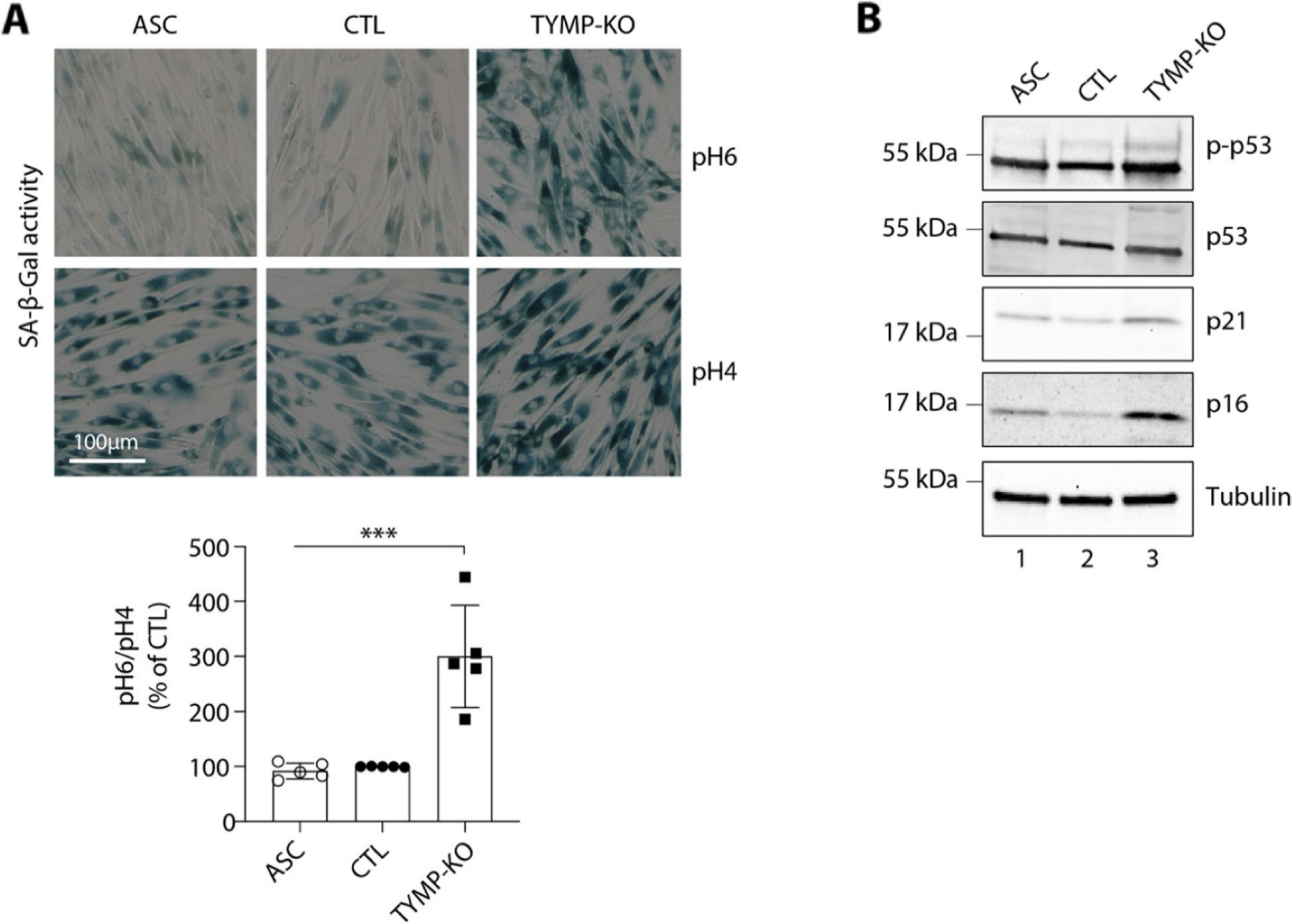
TP deficiency causes oxidative stress and cellular senescence in ASC cells. Data were obtained at D0 in undifferentiated ASC, ASC with a CRISPR-Cas9-mediated TP-knockout (KO), and ASC with a Cas9/scramble gRNA KO corresponding to control (CTL) cells. All results are expressed as means ± SEM of five independent experiments. **A** Representative images of senescence-associated β-galactosidase activity (SA-β-gal) after staining at pH4 and pH6. Scale bar is 100 μm. The SA-β-gal staining ratio at pH 6.0/pH 4.0 was calculated. ***p < 0.001. **B** Evaluation of cellular senescence by Western blotting using antibodies against the indicated proteins. Numbers on the left correspond to molecular weight markers (kDa)

As increase of ROS levels has been associated with an induction of cellular senescence [33], which may result in adipose tissue dysfunction [34–36], we investigated if the defect in adipogenesis seen in TP KO cells might be related to an increased senescence. Compared to WT and control ASC, TP KO cells at D0 were characterized by a 3-fold increase in senescence-associated β-galactosidase (SA-β-gal) activity (*p* < 0.001) (Fig. 6A and Additional file 4: Fig. S6). Enhanced levels of phosphorylated P53 were observed in KO cells (Fig. 6B and Additional file 4: Fig. S6), further underlining the senescent cellular phenotype. In parallel, the levels of P21 and P16, two cell cycle cyclin-dependent kinase inhibitors were significantly increased in KO cells, consistent with increased senescence (Fig. 6B and Additional file 4: Fig. S6). Altogether, these data strongly argue for a functional link between TP dysfunction and cellular senescence in adipocytes.

## Discussion

This translational study identifies biallelic *TYMP* pathogenic variants as a new genetic cause of monogenic lipoatrophic diabetes due to mitochondrial dysfunction. Our data also demonstrate the key role of TP in adipocytes.

MNGIE is characterized by a complex clinical picture involving multiple organs to differing extents in different individuals. The onset of MNGIE disease is usually between the first and second and decade of life [8], as seen in the patients reported herein. Based on a review of the literature, Pacitti et al. proposed a classification of the major and minor MNGIE clinical features [10]. Major signs included severe gastrointestinal dysmotility, cachexia, peripheral neuropathy, ocular symptoms, and diffuse leukoencephalopathy. Minor clinical criteria comprised neurological, muscular, cardiac, and endocrine features. The originality of the current study is to report an inaugural presentation of the disease in the form of isolated lipoatrophic diabetes, thereby showing the heterogeneous clinical onset of MNGIE. In patients from families A and B, generalized lipoatrophy indeed appeared several years before the first gastrointestinal or neurological manifestations. Consequently, the extreme thinness observed in so many patients with MNGIE could correspond to both lipoatrophy due to a primitive adipose tissue dysfunction like in the patients investigated herein, to cachexia secondary to gastrointestinal features as described in other cases, or to a combination of the two. It might seem curious that the generalized lipoatrophic phenotype present in the three patients investigated herein appeared in adolescence. Indeed, the historical classification of lypodystrophies distinguishes the generalized forms with a generalized fat loss apparent at birth, and the partial forms of lipodystrophic syndromes beginning later in life, frequently in adolescence. However, this dichotomy does not apply to all situations and some patients with a genetically defined lipodystrophic syndrome have already been reported with normal fat distribution at birth and appearance of generalized fat loss later in life [37]. In addition, in MNGIE syndrome, the disease phenotype is known to appear progressively, when a threshold level of mutated mtDNA is reached, which is generally when more than 80–90% of total mitochondria are affected [38, 39]. This threshold effect very likely contributes to the protracted interval before the condition manifests and to the disease phenotypic heterogeneity.

We undertook a systematic search in the literature to identify patients with a genetic diagnosis of MNGIE and manifestations of LD. To the best of our knowledge, lipoatrophy has never been reported, though several reports described patients with metabolic alterations evocative of LD. Hypertriglyceridemia was mentioned in a few studies [8, 40–44]. Liver steatosis or cirrhosis associated with hepatomegaly and increased liver enzymes were described in several reports [8, 40–43, 45–50]. Early diabetes was mentioned in a few patients [8, 40, 51–53], although its etiology has never been investigated. In one patient, the disease was reported to start by metabolic manifestations including diabetes, hypertriglyceridemia, and liver steatosis [54], which is reminiscent of current observations. All these data show that the metabolic part of the MNGIE clinical spectrum might be underestimated.

The patients investigated herein carry in the homozygous state two novel *TYMP* molecular defects, including a splice site and a missense variant, whose pathogenicity was confirmed by biochemical assays. This study shows that *TYMP* analysis should enter genetic routine diagnosis of monogenic lipoatrophic diabetes. *TYMP* pathogenic variants are found in ethnically diverse populations [10, 55]. It is currently not possible to state the prevalence of MNGIE as the disorder is probably underdiagnosed due to its multisystem presentation [54]. The condition is not familiar to a majority of clinicians, and patients frequently undergo referral to several different specialties over a protracted period of time before a diagnosis is achieved. This work should increase clinical awareness of the clinical heterogeneity and atypical presentations of MNGIE, thereby reducing diagnostic delay and improving patient care since management of MNGIE requires the coordinated effort of different clinical specialties. Genetic counseling is fundamental in this autosomal recessive disease and prenatal diagnosis should be proposed, with a 25% risk for offspring of carrier parents to be affected.

A few mice models of MNGIE syndrome have been generated over the last past 20 years [56–58]. They were used to study if the pathogenesis of MNGIE involving mitochondrial DNA defects could be attributable to aberrant thymidine metabolism [56], to characterize the biochemical, genetic and histological features of MNGIE [57], and to study the role of deoxynucleoside accumulation in the pathogenesis of MNGIE [58]. Several limitations were encountered when using these animal models. In the mouse, thymidine is not only phosphorylated by thymidine phosphorylase, but also by uridine phosphorylase 1 and uridine phosphorylase 2. In contrast, in the human, thymidine is solely metabolized by thymidine phosphorylase. To partially circumvent this problem, authors established murine models based on double KO of *Tymp*^−/−^ and *Upp1*^−/−^ genes, but to the best of our knowledge, the triple KO has not been generated to date. Consequently, KO animals have a 10-fold increase in plasma thymidine and deoxyuridine, compared to a more than 100-fold increase in the human. Mice models only display minor cerebral signs, but no gastrointestinal or skeletal muscle involvement. This might be explained by the lower increase in deoxyribonucleoside levels, by the fact that mice may not live long enough to accumulate sufficient mtDNA damage, or by a potentially stronger impact of deoxyribonucleoside imbalance in humans. In order to recreate the phenotype of MNGIE in this model the exogenous administration of thymidine and deoxyuridine by dietary supplementation is required [58].

TP was originally identified as platelet-derived endothelial cell growth factor (PDECGF), an angiogenic factor [59]. TP, which is found in a wide range of normal tissues, plays an important role in angiogenesis [60], as well as inflammation [61, 62]. The protein is highly expressed in many types of cancers, including the lung and breast [29, 63, 64]. Nevertheless, more than 30 years after the identification of the TP protein, its multifaceted role is far from being elucidated. In addition, tissue-specific models of MNGIE relevant to organs affected in this syndrome are scarce [65], and besides the nervous and enteric system, the cellular consequences of the lack of TP deficiency are not well addressed. In particular, there is no data on the role of *TYMP* in the adipose tissue and we are the first to demonstrate that TP is present in adipocytes. CRISPR-Cas9 KO of TP in ASC led to a major defect in adipocyte differentiation and function, with a major decrease in intra-cellular lipid levels, triglyceride content, and decreased expression of adipogenesis and mature adipocyte markers. Insulin signaling was also altered, even in pre-adipocytes. These data are consistent with the lipoatrophic and insulin-resistant phenotype of the patients investigated herein. Such an adipocyte differentiation defect has been reported in other lipoatrophic diabetes of various genetic origins [66–68]. Lipoatrophic diabetes are indeed characterized by an incapacity of adipose tissue to store triglycerides, leading to ectopic fat depots and severe insulin resistance. The profound serum leptin and adiponectin deficiency observed in patients further confirms an endocrine defect of adipose tissue, since these hormones are secreted by mature adipocytes.

What is the cellular link between the loss of TP activity and adipogenesis defect? The current study demonstrates the key role of TP for mitochondrial homeostasis and associated adipocyte differentiation and functions. TP KO in ASC induce oxidative stress with a high rate of ROS production, associated with altered mitochondrial respiration. Recent reports emphasize the importance of mitochondria in white adipose tissue biology. In addition to their crucial role in energy homeostasis, mitochondria are the main site of ROS generation. When moderately produced, ROS function as physiological signaling molecules and promote adipocyte differentiation. Primary human mesenchymal stem cells undergoing differentiation into adipocytes indeed display an early increase in mitochondrial metabolism, biogenesis, and ROS generation [69]. However, under different stress conditions, mitochondrial ROS overproduction induces the expression of adipogenic repressors and inhibits adipocyte differentiation [32]. Balanced mROS production is thus at the core of proper metabolic maintenance, and unbalanced mROS production appears as an important trigger of metabolic disorders [70]. Our study provides an additional illustration to these basic research data by the description of a monogenic disease known to induce an accumulation of mitochondrial DNA mutations and leading to major adipocyte dysfunction. In addition to its deleterious effect on adipocyte differentiation, emerging evidence points to ROS overproduction as a direct cause of insulin resistance. As an example, a previous study has shown that adipocytes subjected to oxidative stress induced by chemical agents exhibited an insulin-resistant state [71]. In the same line and more generally, enhanced ROS production is observed in insulin resistance states and clinical diabetes [72]. Hyperglycemia indeed increases the mitochondrial electron transfer chain’s activity until saturation, finally resulting in disruption and uncoupling of several key oxidative reactions and excessive ROS production [72]. This is consistent with our data showing that TP KO undifferentiated ASC do not respond properly to insulin stimulation.

The causal link between ROS, cellular senescence, aging, and senescence-associated pathologies is intensely studied [33]. It is widely assumed that ROS produced by mitochondria are involved in senescence [73–76]. Therefore, we might speculate that the cellular senescence observed in TP KO cells is partly due to mitochondrial dysfunction and ROS overproduction, though direct evidence is lacking. In addition, cellular senescence has already been reported in a few cellular models of lipodystrophic syndromes or in cells from certain patients with LD [34, 35, 77, 78]. More generally, increased cellular senescence has been functionally linked to fat-related metabolic dysfunction, which is underlined by the data of the current study [36].

The role of the mitochondria in monogenic forms of diabetes is beginning to be better understood. In recent years, a few genes involved in monogenic lipoatrophic diabetes and encoding proteins playing a key role for mitochondrial function have been identified. As an example, *MFN2* encoding mitofusin 2 has been involved in the lipodystrophic Launois-Bensaude syndrome [67, 79, 80], and *SLC25A24* encoding a calcium-binding mitochondrial carrier protein has been involved in a complex progeroid syndrome with lipoatrophy [81]. These monogenic lipoatrophic diabetes have to be distinguished from the so-called mitochondrial diabetes due to molecular defects in mitochondrial DNA. Mitochondrial diabetes usually leads to dysfunction of pancreatic islet beta-cells due to their poor ability to resist oxidative stress induced by mitochondrial chain dysfunction and to a consequent defect in insulin secretion [82, 83]. This situation is different from that of the patients investigated herein, who displayed severe insulin resistance and a major demise in adipose tissue, thereby underlining the key role of mitochondrial homeostasis for proper adipose tissue function. The spectrum of genes playing a key role for mitochondrial function and involved in adipose tissue dysfunction should therefore continue to expand in the near future.

## Conclusions

In summary, this study broadens the clinical spectrum associated with *TYMP* pathogenic variants and justifies screening of this gene in insulin-resistant lipoatrophic diabetes. The demonstration of the crucial of TP for adipocyte differentiation and function through the control of mitochondrial homeostasis also highlights the importance of mitochondria in adipose tissue physiology.

## Supplementary Information

**Additional file 1: Table S1.** Prediction of pathogenicity of variants identified in *TYMP*. Bioinformatic tools used to predict the pathogenicity of splice site and missense variants are not the same. CADD: Combined Annotation Dependent Depletion; SIFT: Sorting Intolerant From Tolerant; SPiP: Splicing Prediction Pipeline.

**Additional file 2: Table S2.** List of predicted off-target sequences of the CRISPR/Cas9 editing strategy, with mismatch position and genomic location. The CRISPOR web tool (http://crispor.tefor.net/) is well recognized to predict the risk of off-target sequences by providing a cutting frequency determination (CFD) specificity score ranging from 1 to 100. The higher the number, the lower the risk of off-target effects. It is based on the accurate CFD off-target model from Doench JG et al. (Nat Biotechnol 2016 Feb;34(2):184-196), which recommends guides with a CFD specificity score > 50. The gRNA used herein to target *TYMP* exon 5 has a CFD score of 84. This gRNA did not match perfectly any other genomic region. The table below provides a list of potential off-target sequences with up to three mismatches with the gRNA used (CAGAGATGTGACAGCCACCG). Notably, off-targets are considered if they are flanked by an NGG motif, which corresponds to the PAM sequence allowing the Cas9 to cut DNA.

**Additional file 3.** Uncropped WBs: Uncropped and unedited Western blots seen in the different figures.

**Additional file 4: Fig. S1.** RNA expression of *TYMP* within individual tissues. Data were extracted from the GTex Portal (https://gtexportal.org), a web resource studying human gene expression and regulation, and sorted by decreasing expression rate. The V8 Release used for this analysis was based on data from 17382 samples, 54 tissues, and 948 donors. Tissues with a TPM value under 40 were not presented. TPM: transcripts per million. **Fig. S2.** Assessment of CRISPR-Cas9 editing efficiency with gRNA targeting *TYMP* exon 5 in control and edited ASC. (A) Sanger sequencing of the target region (exon 5) confirmed high level of recombination. The expected break site is indicated by a vertical dotted line and the gRNA sequence is underlined. (B) Determination of CRISPR indel pattern in control and edited ASC by analyzing Sanger sequencing data with the Synthego software (https://ice.synthego.com). Left panel: The discordance plot details the rate of sequence alignment per base between the control and edited samples in the inference window (i.e., the region around the recombination site). Before the gRNA target site, the green line (edited sample) and the orange line (control sample) are close together. After the gRNA target site, a jump is observed, corresponding to a high level of sequence misalignment. Right panel: The graph displays the frequency of indels in relationship with the indel size. The editing efficiency corresponding to the recombination rate was evaluated at 91%. **Fig. S3.** Quantification of osteogenic markers in control and edited ASC. Data were obtained in ASC, ASC with a CRISPR-Cas9-mediated TP-knockout (KO), and ASC transduced with a Cas9/scramble gRNA plasmid corresponding to control (CTL) cells. The Western blots of osteoblast markers related to Fig. 3G were quantified using FIJI software and normalized to the value of CTL cells at D14. *p*-values were determined by analysis of variance (ANOVA) with Bonferroni’s *post hoc* multiple comparison test. n.s., non-significant. **Fig. S4.** Quantification of adipocyte markers in control and edited ASC. Data were obtained in ASC, ASC with a CRISPR-Cas9-mediated TP-knockout (KO), and ASC transduced with a Cas9/scramble gRNA plasmid corresponding to control (CTL) cells. The Western blots of adipocyte markers related to Fig. 4A were quantified using FIJI software and normalized to the value of CTL cells at D20. *p*-values were determined by analysis of variance (ANOVA) with Bonferroni’s *post hoc* multiple comparison test. *p < 0.05, **p < 0.01, ***p < 0.001, ****p < 0.0001. **Fig. S5.** Quantification of insulin signaling markers in control and edited ASC. Data were obtained in ASC, ASC with a CRISPR-Cas9-mediated TP-knockout (KO), and ASC transduced with a Cas9/scramble gRNA plasmid corresponding to control (CTL) cells. (A) The Western blots of insulin markers related to Fig. 4B were quantified using FIJI software and normalized to the value of CTL cells at D20. (B) The Western blots of insulin markers related to Fig. 4C were quantified using FIJI software and normalized to the value of CTL cells at D0. *p*-values were determined by analysis of variance (ANOVA) with Bonferroni’s *post hoc* multiple comparison test. **p < 0.01, ***p < 0.001, ****p < 0.0001, n.s., non-significant. **Fig. S6.** Quantification of senescence markers in control and edited ASC. Data were obtained in ASC, ASC with a CRISPR-Cas9-mediated TP-knockout (KO), and ASC transduced with a Cas9/scramble gRNA plasmid corresponding to control (CTL) cells. The Western blots of senescence markers related to Fig. 6B were quantified using FIJI software and normalized to the value of CTL cells at D0. *p*-values were determined by analysis of variance (ANOVA) with Bonferroni’s *post hoc* multiple comparison test. **p < 0.01, ****p < 0.0001.

## Data Availability

Further information and requests for resources and reagents should be directed to and will be fulfilled by J. Gautheron (jeremie.gautheron@inserm.fr) and I. Jéru (isabelle.jeru@aphp.fr).

## Abbreviations

ACMG: American College of Medical Genetics and Genomics
ALT: Alanine aminotransferase
ANOVA: One-way analysis of variance
ASC: Adipose stem cell
AST: Aspartate aminotransferase
β-gal: β-galactosidase
BMI: Body mass index
C/EBPα: CCAAT/enhancer-binding protein-alpha
CM-H_2_DCFHDA: 2,7-Dichlorodihydrofluorescein diacetate
CTL: Control
DXA: Dual-energy X-ray absorptiometry
ECLip: European Consortium of Lipodystrophies
ERK: Extracellular-regulated kinase
FAS: Fatty acid synthase
FCCP: Carbonylcyanide-p-trifluoromethoxy-phenylhydrazone
FCS: Fetal calf serum
FGF: Fibroblast growth factor
FSH: Follicle-stimulating hormone
GGT: Gamma glutamyl transpeptidase
gRNA: Guide RNA
HbA1c: Glycated hemoglobin
HDL: High-density lipoprotein
IBMX: 3-Isobutyl-1-methylxanthine
IGI: Insulinogenic index
IRβ: Receptor β subunit
IRS1: Insulin receptor substrate-1
KO: Knock-out
LD: Lipoatrophic diabetes
LH: Luteinizing hormone
MODY: Maturity-onset diabetes of the young
MNGIE: Mitochondrial neurogastrointestinal encephalomyopathy
MRI: Magnetic resonance imaging
mROS: Mitochondrial ROS
mtDNA: Mitochondrial DNA
OCR: Oxygen consumption rate
OGTT: Oral glucose tolerance test
PBS: Phosphate-buffered saline
PFA: Paraformaldehyde
PPARγ: Peroxisome proliferator-activated receptor gamma
P/S: Penicillin/streptomycin
SA-β-gal: Senescence-associated β-galactosidase
SD: Standard deviation
SDS-PAGE: Sodium dodecyl sulfate - polyacrylamide gel electrophoresis
TP: Thymidine phosphorylase
WES: Whole exome sequencing
WT: Wild type
