# Supplementary material for: Loss of thymidine phosphorylase activity disrupts adipocyte differentiation and induces insulin-resistant lipoatrophic diabetes
Source: BMC Med. 2022 Mar 28;20:95. doi: 10.1186/s12916-022-02296-2 (PMC8958798; doi:10.1186/s12916-022-02296-2)
Supplement: Supplementary file 1 — Additional file 1: Table S1. Prediction of pathogenicity of variants identified in TYMP. Bioinformatic tools used to predict the pathogenicity of splice site and missense variants are not the same. CADD: Combined Annotation Dependent Depletion; SIFT: Sorting Intolerant From Tolerant; SPiP: Splicing Prediction Pipeline. [file 12916_2022_2296_MOESM1_ESM.docx]

**Additional file 1: Table S1. Prediction of pathogenicity of variants identified in *TYMP***.

Bioinformatic tools used to predict the pathogenicity of splice site and missense variants are not the same. CADD: Combined Annotation Dependent Depletion; SIFT: Sorting Intolerant From Tolerant; SPiP: Splicing Prediction Pipeline.

| ***TYMP* : c.647-1G>A** | | | |
| --- | --- | --- | --- |
| **Bioinformatic tool** | **Website address** | **Prediction of pathogenicity** | **Description – Threshold of scores to predict pathogenicity** |
| spliceAI | https://github.com/Illumina/SpliceAI | Loss of splicing acceptor site  Score: 0.94 | Threshold > 0.8 for deleterious impact |
| SPiP | https://github.com/raphaelleman/SPiP | High risk of alteration of the consensus splice site: 98.41 % [91.47 % - 99.96 %] | Prediction of the risk for the variant to alter splicing |
| CADD | https://cadd.gs.washington.edu/snv | CADD Phred score: 34.0 | Ranking score with no given cut-off, higher scores being more likely to be deleterious.  Scores above 20 are usually considered as “likely deleterious” |
| ***TYMP* : c.392C>T** | | | |
| **Bioinformatic tool** | **Website adress** | **Prediction of pathogenicity** | **Description – Threshold of scores to predict pathogenicity** |
| PolyPhen-2 | http://genetics.bwh.harvard.edu/pph2/ | “Probably Damaging” variant  Score: 1.0 | Threshold > 0.447 and 0.909 for “Possibly Damaging” and “Probably Damaging” variants, respectively |
| REVEL | https://sites.google.com/site/revelgenomics/ | “Damaging” variant  Score: 0.853 | Threshold > 0.5 for “Damaging” variants |
| SIFT | https://sift.bii.a-star.edu.sg/ | “Damaging” variant  Score: 0.0 | Threshold < 0.05 for “Damaging” variants |
| CADD | https://cadd.gs.washington.edu/snv | CADD Phred score : 28.7 | Ranking score with no given cut-off, higher scores being more likely to be deleterious.  Scores above 20 are usually considered as “likely deleterious” |
