# Supplementary material for: Loss of thymidine phosphorylase activity disrupts adipocyte differentiation and induces insulin-resistant lipoatrophic diabetes
Source: BMC Med. 2022 Mar 28;20:95. doi: 10.1186/s12916-022-02296-2 (PMC8958798; doi:10.1186/s12916-022-02296-2)
Supplement: Supplementary file 2 — Additional file 2: Table S2. List of predicted off-target sequences of the CRISPR/Cas9 editing strategy, with mismatch position and genomic location. The CRISPOR web tool (http://crispor.tefor.net/) is well recognized to predict the risk of off-target sequences by providing a cutting frequency determination (CFD) specificity score ranging from 1 to 100. The higher the number, the lower the risk of off-target effects. It is based on the accurate CFD off-target model from Doench JG et al. (Nat Biotechnol 2016 Feb;34(2):184-196), which recommends guides with a CFD specificity score > 50. The gRNA used herein to target TYMP exon 5 has a CFD score of 84. This gRNA did not match perfectly any other genomic region. The table below provides a list of potential off-target sequences with up to three mismatches with the gRNA used (CAGAGATGTGACAGCCACCG). Notably, off-targets are considered if they are flanked by an NGG motif, which corresponds to the PAM sequence allowing the Cas9 to cut DNA. [file 12916_2022_2296_MOESM2_ESM.docx]

**Additional file 2: Table S2. List of predicted off-target sequences of the CRISPR/Cas9 editing strategy, with mismatch position and genomic location.** The CRISPOR web tool (<http://crispor.tefor.net/>) is well recognized to predict the risk of off-target sequences by providing a cutting frequency determination (CFD) specificity score ranging from 1 to 100. The higher the number, the lower the risk of off-target effects. It is based on the accurate CFD off-target model from Doench JG et al. (Nat Biotechnol 2016 Feb;34(2):184-196), which recommends guides with a CFD specificity score > 50. The gRNA used herein to target *TYMP* exon 5 has a CFD score of 84. This gRNA did not match perfectly any other genomic region. The table below provides a list of potential off-target sequences with up to three mismatches with the gRNA used (CAGAGATGTGACAGCCACCG). Notably, off-targets are considered if they are flanked by an NGG motif, which corresponds to the PAM sequence allowing the Cas9 to cut DNA.

| **Number of mismatches** | **Potential off-target sequences (mismatches are in red and bold characters)** | **Locus of the off-target**  **(location, gene)** |
| --- | --- | --- |
| 2 | CAGA**C**ATGTGACAGCCAC**T**G | intergenic |
| 2 | CAGAG**C**TGTGACAGCCACC**T** | intergenic |
| 3 | CAGAG**T**TGTGACA**A**CCACC**A** | intergenic |
| 3 | CAGA**C**ATGTG**G**CAGCCACC**A** | intronic, *TFAP2E* |
| 3 | CAG**G**GATG**G**GACAGC**A**ACCG | intronic, *ARHGEF10* |
| 3 | CAG**G**GATG**G**GAC**G**GCCACCG | intronic, *VSX2* |
| 3 | CAGAG**C**TGTG**G**CAGCCACC**T** | intergenic |
| 3 | CA**A**AGAT**T**TGACAGCCAC**A**G | intergenic |
| 3 | CAGAG**C**TG**A**GACAGCCACC**C** | exonic, *SERPINA4/SERPINA5* |
| 3 | **G**AGAGA**A**GTGACAGC**G**ACCG | intergenic |
| 3 | CAGA**CC**TGTGAC**T**GCCACCG | intronic, *NEK6* |
| 3 | CA**T**AGATGTGA**G**AGCCAC**A**G | intergenic |
| 3 | CAGAGATGTGACAG**AA**ACC**C** | intergenic |
| 3 | CAGAGA**A**GTG**G**CAGCCA**G**CG | exonic, *LHFPL4* |
| 3 | CA**A**AGATGTGA**A**AGCCA**G**CG | intergenic |
| 3 | CAGAGATGTGACA**T**CCA**AT**G | intronic |
| 3 | CAGAGA**A**GTGACAGCCA**GA**G | intergenic |
| 3 | CAGAGATGTGACA**A**CCA**GA**G | intergenic |
| 3 | CAGAGATGTG**T**C**G**GCC**T**CCG | intergenic |
| 3 | C**T**G**G**GATGTGACAGCCACC**T** | intronic, *LINC01508* |
| 3 | CAGAGATGTGACAG**A**CA**GG**G | intronic, *AP5M1* |
