# Supplementary material for: Loss of thymidine phosphorylase activity disrupts adipocyte differentiation and induces insulin-resistant lipoatrophic diabetes
Source: BMC Med. 2022 Mar 28;20:95. doi: 10.1186/s12916-022-02296-2 (PMC8958798; doi:10.1186/s12916-022-02296-2)
Supplement: Supplementary file 4 — Additional file 4: Fig. S1. RNA expression of TYMP within individual tissues. Data were extracted from the GTex Portal (https://gtexportal.org), a web resource studying human gene expression and regulation, and sorted by decreasing expression rate. The V8 Release used for this analysis was based on data from 17382 samples, 54 tissues, and 948 donors. Tissues with a TPM value under 40 were not presented. TPM: transcripts per million. Fig. S2. Assessment of CRISPR-Cas9 editing efficiency with gRNA targeting TYMP exon 5 in control and edited ASC. (A) Sanger sequencing of the target region (exon 5) confirmed high level of recombination. The expected break site is indicated by a vertical dotted line and the gRNA sequence is underlined. (B) Determination of CRISPR indel pattern in control and edited ASC by analyzing Sanger sequencing data with the Synthego software (https://ice.synthego.com). Left panel: The discordance plot details the rate of sequence alignment per base between the control and edited samples in the inference window (i.e., the region around the recombination site). Before the gRNA target site, the green line (edited sample) and the orange line (control sample) are close together. After the gRNA target site, a jump is observed, corresponding to a high level of sequence misalignment. Right panel: The graph displays the frequency of indels in relationship with the indel size. The editing efficiency corresponding to the recombination rate was evaluated at 91%. Fig. S3. Quantification of osteogenic markers in control and edited ASC. Data were obtained in ASC, ASC with a CRISPR-Cas9-mediated TP-knockout (KO), and ASC transduced with a Cas9/scramble gRNA plasmid corresponding to control (CTL) cells. The Western blots of osteoblast markers related to Fig. 3G were quantified using FIJI software and normalized to the value of CTL cells at D14. p-values were determined by analysis of variance (ANOVA) with Bonferroni’s post hoc multiple comparison test. n.s., non-si [file 12916_2022_2296_MOESM4_ESM.docx]

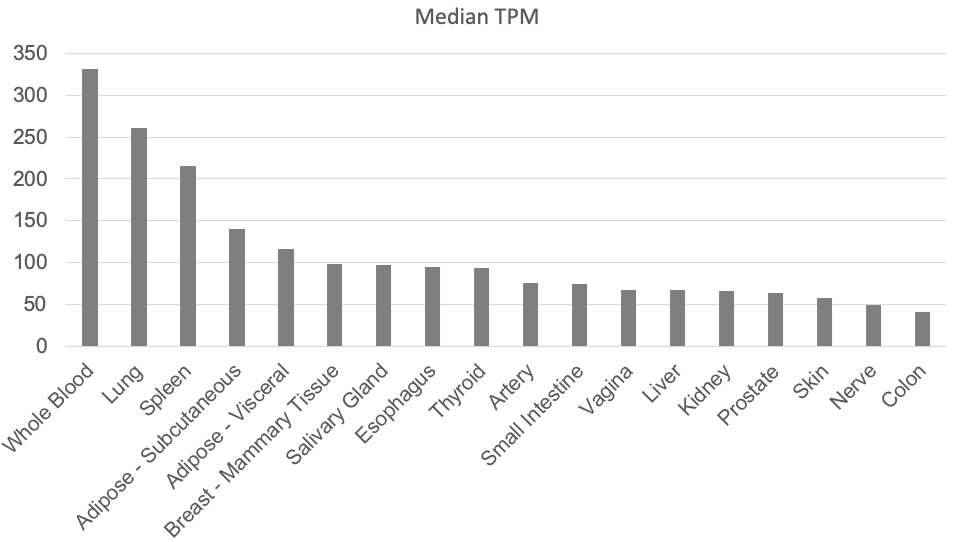


**Fig. S1. RNA expression of *TYMP* within individual tissues.** Data were extracted from the GTex Portal (<https://gtexportal.org>), a web resource studying human gene expression and regulation, and sorted by decreasing expression rate. The V8 Release used for this analysis was based on data from 17382 samples, 54 tissues, and 948 donors. Tissues with a TPM value under 40 were not presented. TPM: transcripts per million.

**
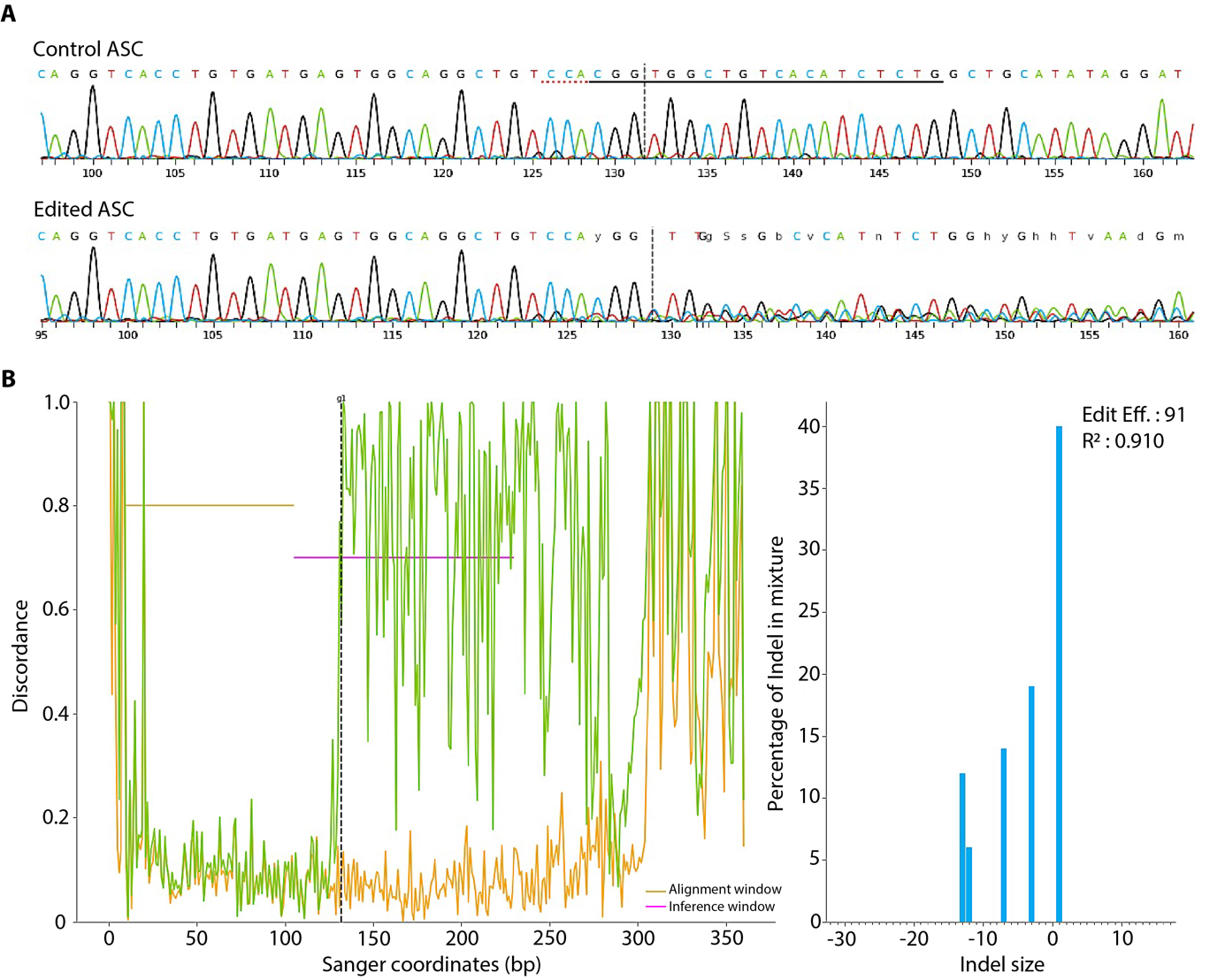
**

**Fig. S2. Assessment of CRISPR-Cas9 editing efficiency with gRNA targeting *TYMP* exon 5 in control and edited ASC.** (**A**) Sanger sequencing of the target region (exon 5) confirmed high level of recombination. The expected break site is indicated by a vertical dotted line and the gRNA sequence is underlined. (**B**) Determination of CRISPR indel pattern in control and edited ASC by analyzing Sanger sequencing data with the Synthego software (<https://ice.synthego.com>). Left panel: The discordance plot details the rate of sequence alignment per base between the control and edited samples in the inference window (i.e., the region around the recombination site). Before the gRNA target site, the green line (edited sample) and the orange line (control sample) are close together. After the gRNA target site, a jump is observed, corresponding to a high level of sequence misalignment. Right panel: The graph displays the frequency of indels in relationship with the indel size. The editing efficiency corresponding to the recombination rate was evaluated at 91%.


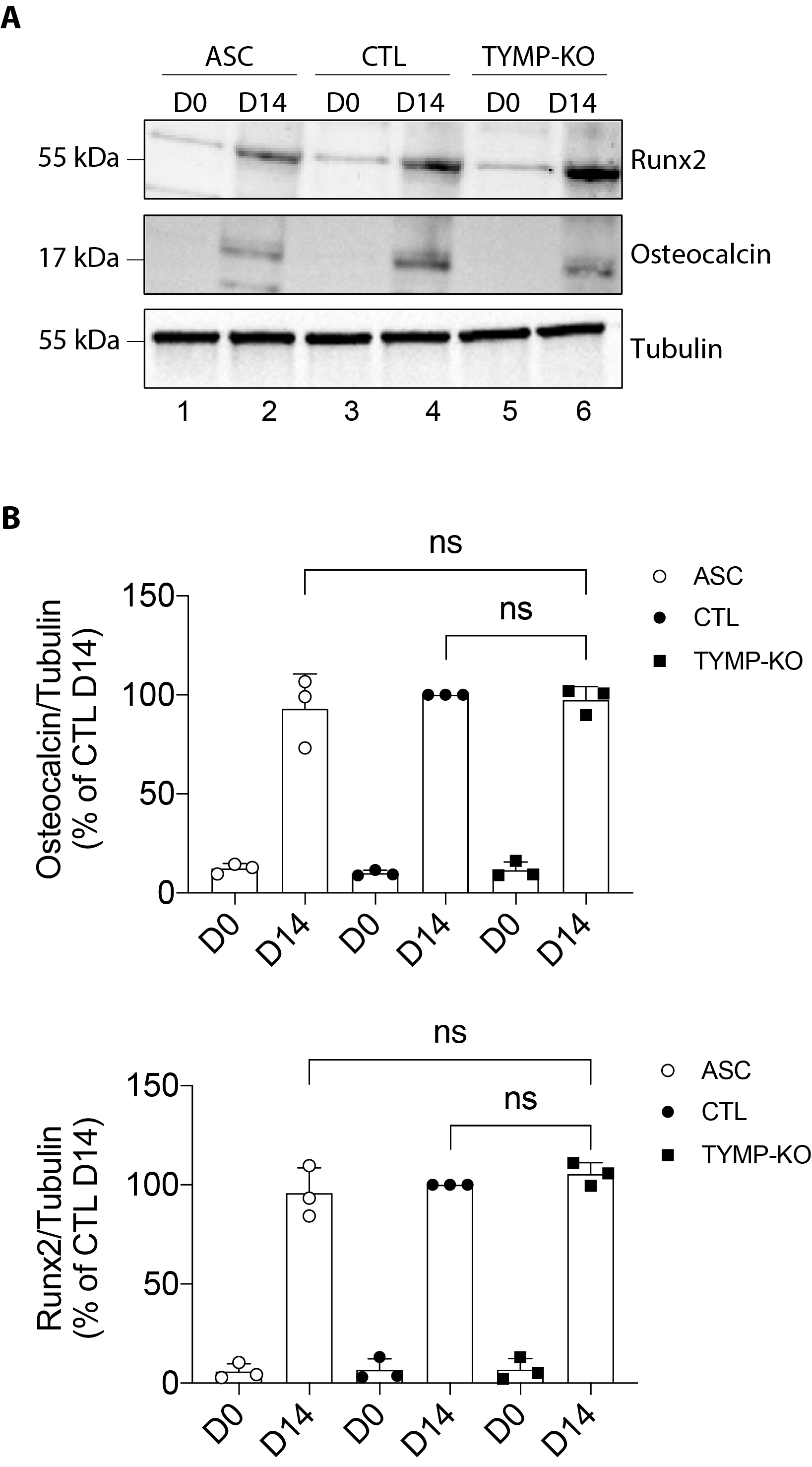


**Fig. S3. Quantification of osteogenic markers in control and edited ASC.** Data were obtained in ASC, ASC with a CRISPR-Cas9-mediated TP-knockout (KO), and ASC transduced with a Cas9/scramble gRNA plasmid corresponding to control (CTL) cells. The Western blots of adipocyte markers related to Fig. 3G were quantified using FIJI software and normalized to the value of CTL cells at D14. *p*-values were determined by analysis of variance (ANOVA) with Bonferroni’s *post hoc* multiple comparison test. n.s., non-significant.


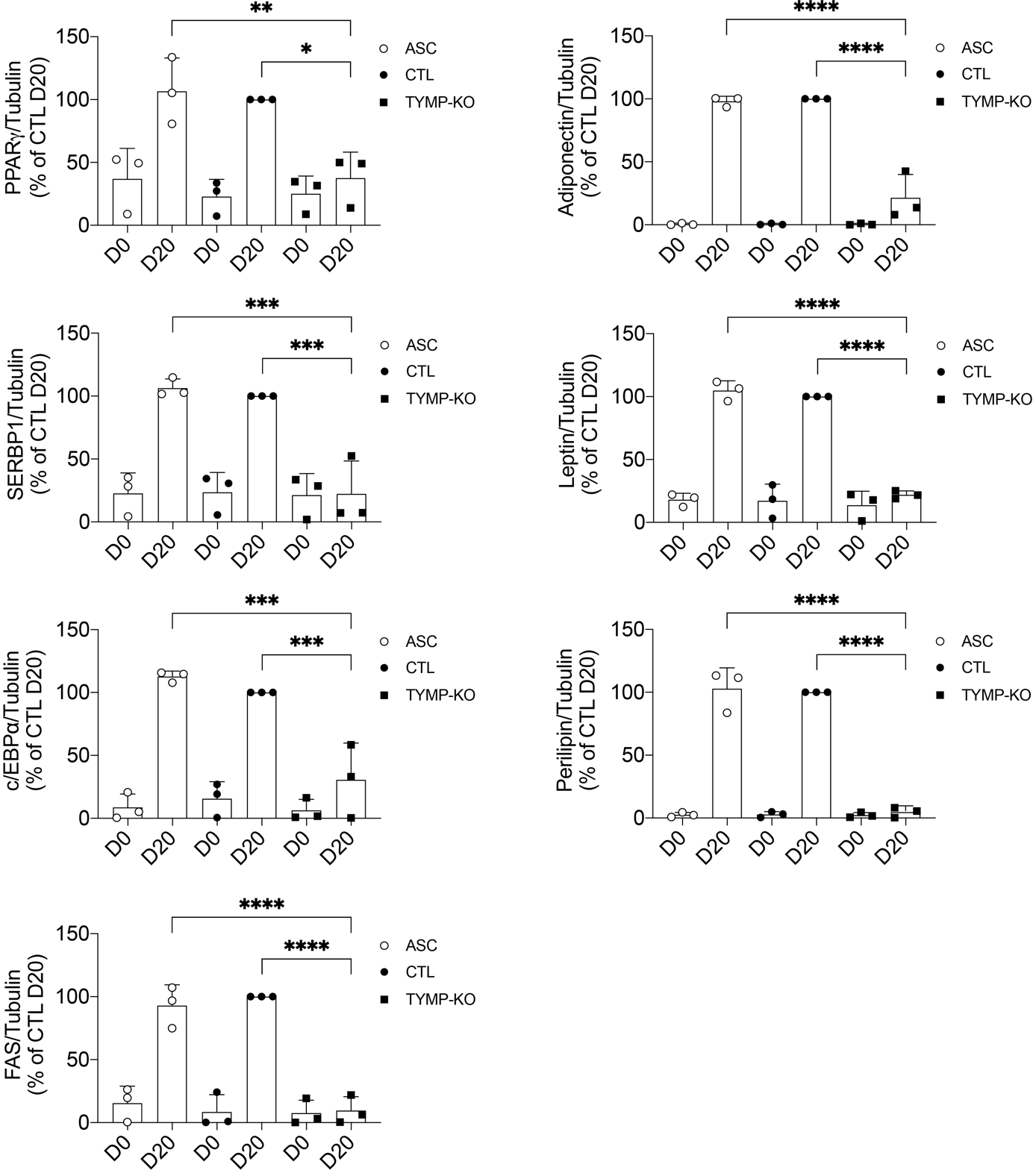


**Fig. S4. Quantification of adipocyte markers in control and edited ASC.** Data were obtained in ASC, ASC with a CRISPR-Cas9-mediated TP-knockout (KO), and ASC transduced with a Cas9/scramble gRNA plasmid corresponding to control (CTL) cells. The Western blots of adipocyte markers related to Fig. 4A were quantified using FIJI software and normalized to the value of CTL cells at D20. *p*-values were determined by analysis of variance (ANOVA) with Bonferroni’s *post hoc* multiple comparison test. **p* < 0.05, ***p* < 0.01, ****p* < 0.001 and *****p* < 0.0001.


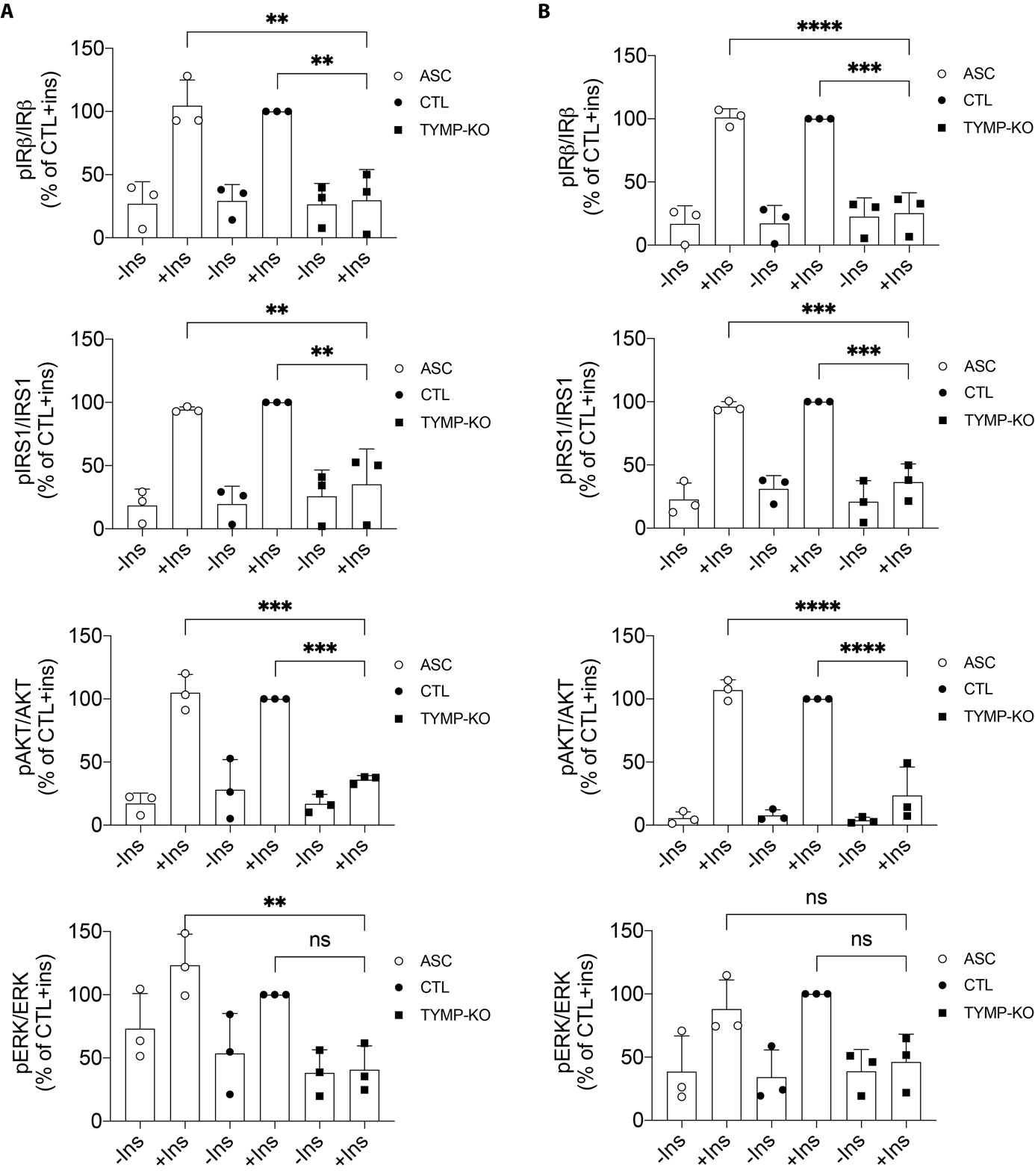


**Fig. S5. Quantification of insulin signaling markers in control and edited ASC.** Data were obtained in ASC, ASC with a CRISPR-Cas9-mediated TP-knockout (KO), and ASC transduced with a Cas9/scramble gRNA plasmid corresponding to control (CTL) cells. **(A)** The Western blots of insulin markers related to Fig. 4B were quantified using FIJI software and normalized to the value of CTL cells at D20. **(B)** The Western blots of insulin markers related to Fig. 4C were quantified using FIJI software and normalized to the value of CTL cells at D0. *p*-values were determined by analysis of variance (ANOVA) with Bonferroni’s *post hoc* multiple comparison test. ***p* < 0.01, ****p* < 0.001, *****p* < 0.0001, n.s., non-significant.


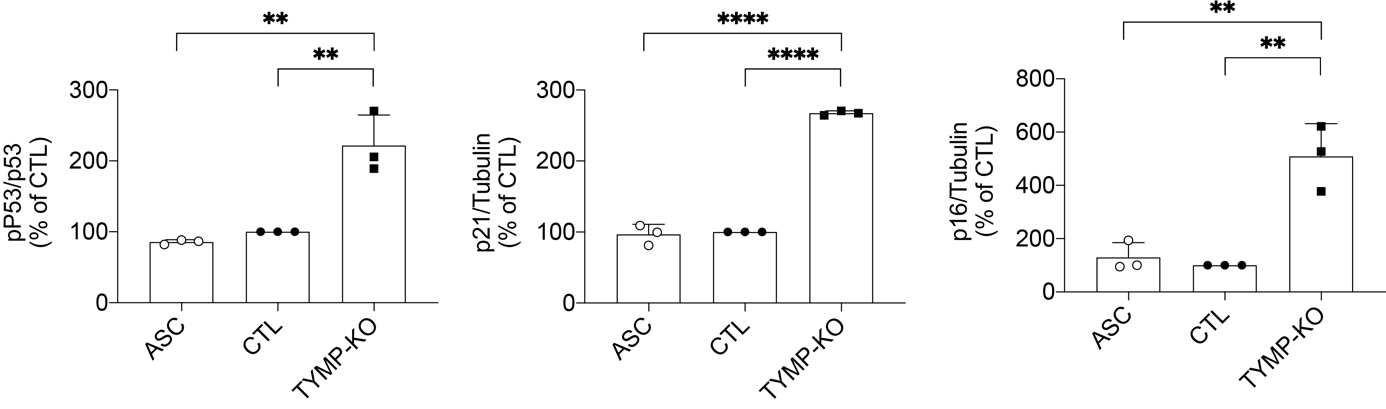


**Fig. S6. Quantification of senescence markers in control and edited ASC.** Data were obtained in ASC, ASC with a CRISPR-Cas9-mediated TP-knockout (KO), and ASC transduced with a Cas9/scramble gRNA plasmid corresponding to control (CTL) cells. The Western blots of senescence markers related to Fig. 6B were quantified using FIJI software and normalized to the value of CTL cells at D0. *p*-values were determined by analysis of variance (ANOVA) with Bonferroni’s *post hoc* multiple comparison test. ***p* < 0.01 and *****p* < 0.0001.
