# Supplementary material for: Loss of thymidine phosphorylase activity disrupts adipocyte differentiation and induces insulin-resistant lipoatrophic diabetes
Source: BMC Med. 2022 Mar 28;20:95. doi: 10.1186/s12916-022-02296-2 (PMC8958798; doi:10.1186/s12916-022-02296-2)

Figure 3B

anti-TYMP

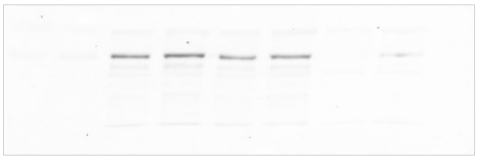

anti-Tubulin

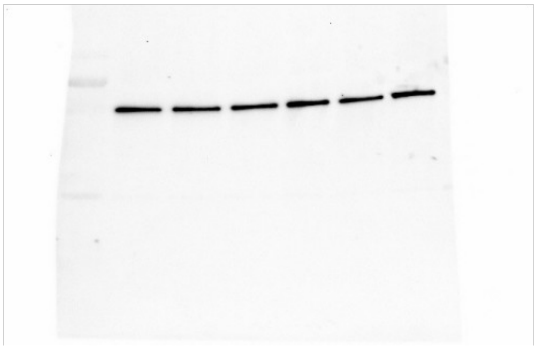

Figure 4A

anti-PPARγ

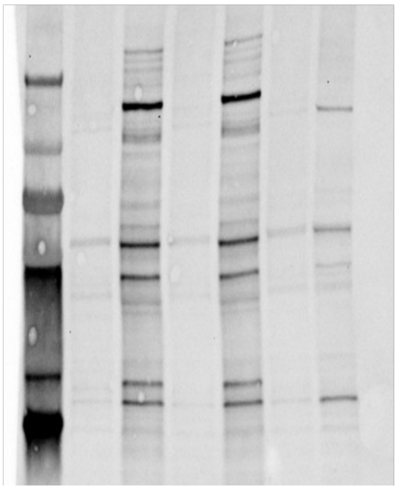

anti-SREBP1

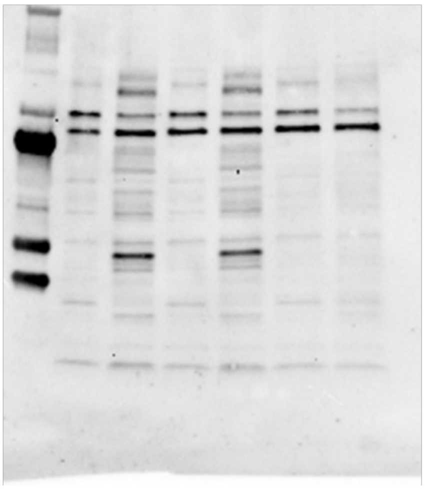

Figure 4A

anti-c/EBPα

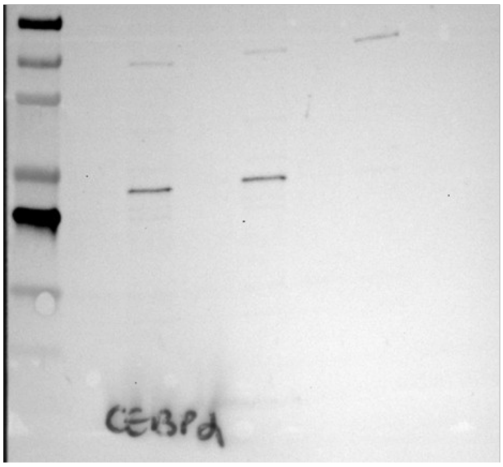

anti-FAS

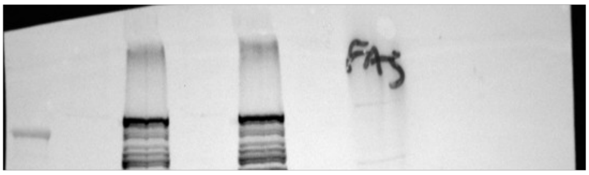

Figure 4A

anti-Adiponectin

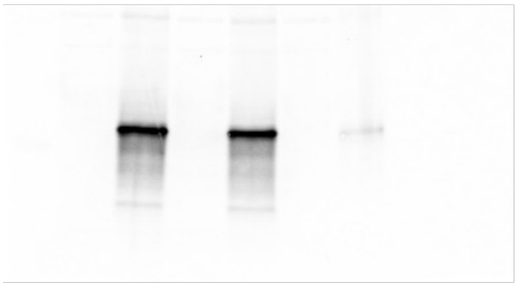

anti-Perilipin

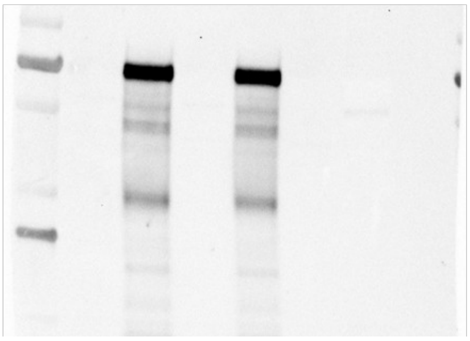

Figure 4A

anti-Leptin

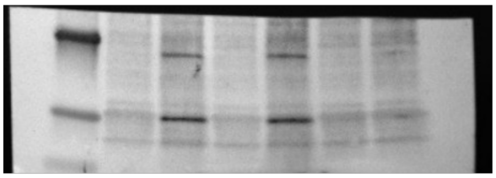

anti-Tubulin

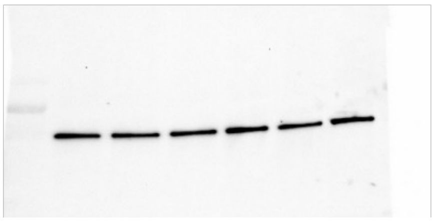

Figure 4B

anti-p-IRβ

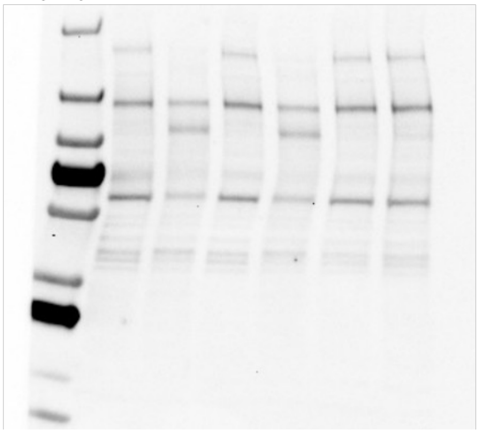

anti-IRβ

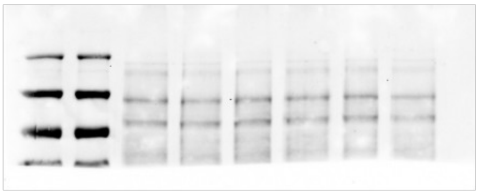

Figure 4B

anti-p-IRS1

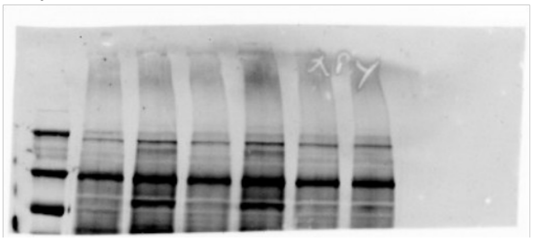

anti-IRS1

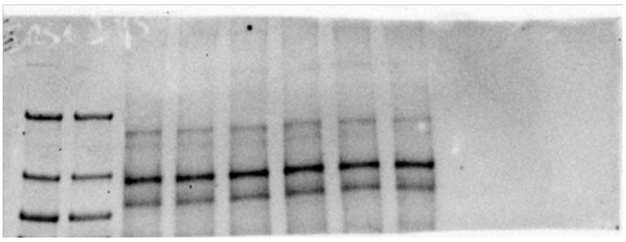

Figure 4B

anti-p-AKT & anti-p-ERK

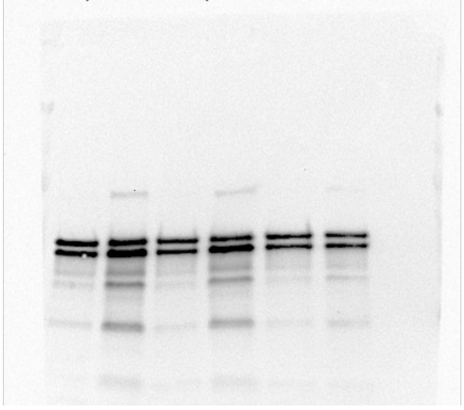

anti-AKT & anti-ERK

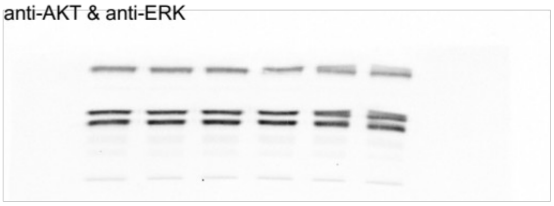

Figure 4C

anti-p-IRβ

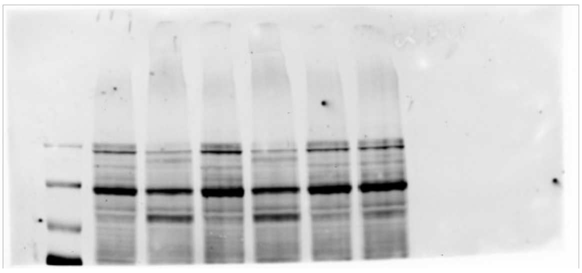

anti-IRβ

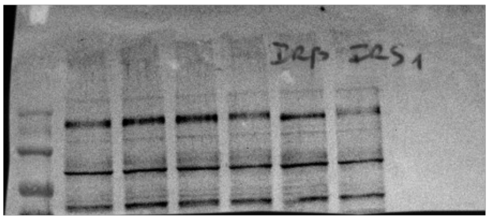

Figure 4C

anti-p-IRS1

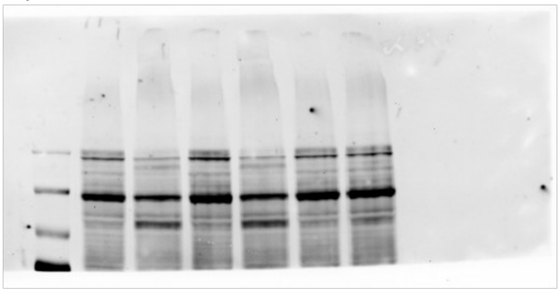

anti-IRS1

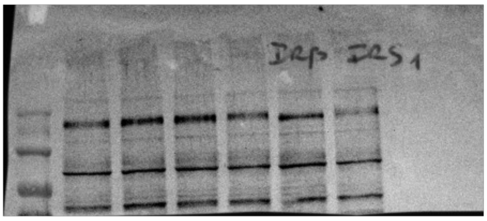

Figure 4C

anti-p-AKT

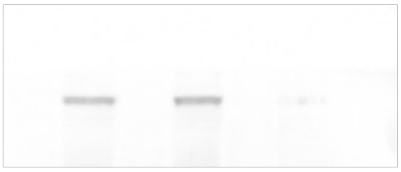

anti-AKT

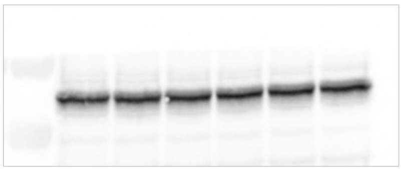

anti-p-ERK

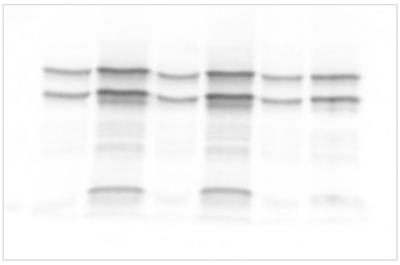

anti-ERK

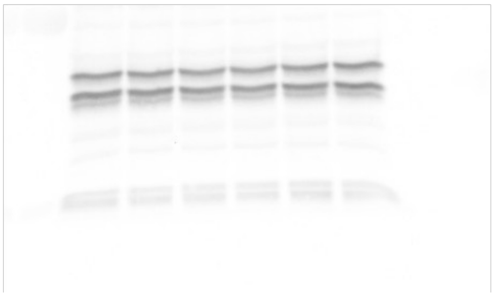

Figure 6B

anti-p-p53

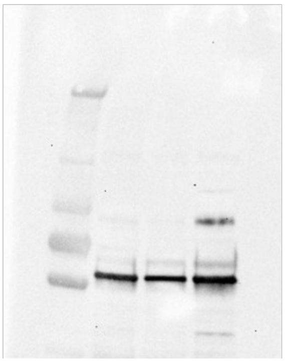

anti-p53

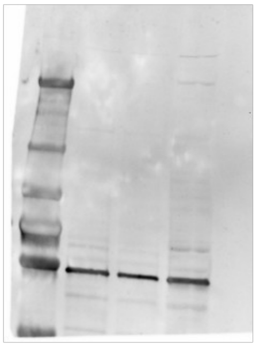

Figure 6B

anti-p21

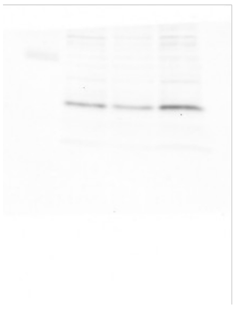

anti-p16

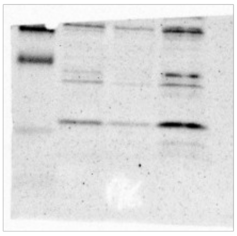

anti-Tubulin

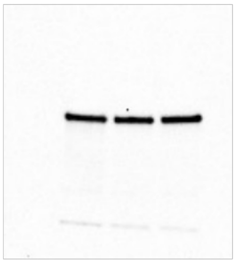

Fig S3

anti-Runx2

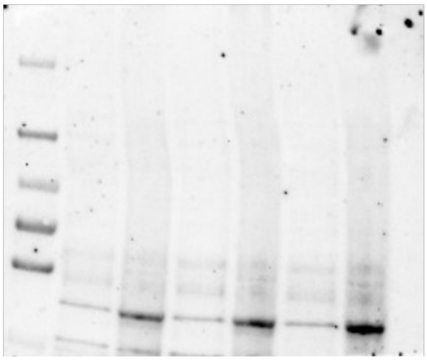

anti-Osteocalcin

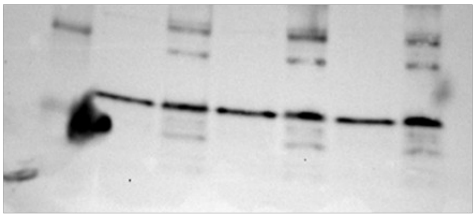

Fig S3

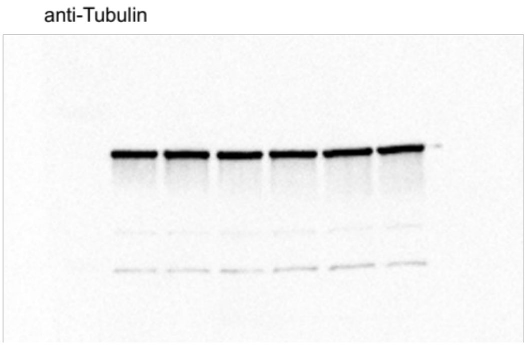

Supplement: Supplementary file 4 [file 12916_2022_2296_MOESM3_ESM.docx]
